# Axonal transport deficits in the pathogenesis of diabetic peripheral neuropathy

**DOI:** 10.3389/fendo.2023.1136796

**Published:** 2023-03-28

**Authors:** Cunqing Yang, Xuefei Zhao, Xuedong An, Yuehong Zhang, Wenjie Sun, Yuqing Zhang, Yingying Duan, Xiaomin Kang, Yuting Sun, Linlin Jiang, Fengmei Lian

**Affiliations:** Department of Endocrinology, Guang’anmen Hospital, China Academy of Chinese Medical Sciences, Beijing, China

**Keywords:** axonal transport, diabetic peripheral neuropathy, diabetes, microtubules, molecular motors, cytoskeleton

## Abstract

Diabetic peripheral neuropathy (DPN) is a chronic and prevalent metabolic disease that gravely endangers human health and seriously affects the quality of life of hyperglycemic patients. More seriously, it can lead to amputation and neuropathic pain, imposing a severe financial burden on patients and the healthcare system. Even with strict glycemic control or pancreas transplantation, peripheral nerve damage is difficult to reverse. Most current treatment options for DPN can only treat the symptoms but not the underlying mechanism. Patients with long-term diabetes mellitus (DM) develop axonal transport dysfunction, which could be an important factor in causing or exacerbating DPN. This review explores the underlying mechanisms that may be related to axonal transport impairment and cytoskeletal changes caused by DM, and the relevance of the latter with the occurrence and progression of DPN, including nerve fiber loss, diminished nerve conduction velocity, and impaired nerve regeneration, and also predicts possible therapeutic strategies. Understanding the mechanisms of diabetic neuronal injury is essential to prevent the deterioration of DPN and to develop new therapeutic strategies. Timely and effective improvement of axonal transport impairment is particularly critical for the treatment of peripheral neuropathies.

## 1 Introduction

Diabetes mellitus (DM) has become a serious global public health problem. According to the International Diabetes Federation (IDF), more than 460 million people worldwide suffer from diabetes mellitus. By 2045, the number of people with diabetes worldwide is expected to reach 628 million (1) The incidence of diabetic complications is also on the rise, with diabetic peripheral neuropathy (DPN) being the most widespread complication of diabetes and a major cause of disability, foot ulcers and even amputation (2–4).The pathogenesis of DPN involves oxidative stress, excessive activation of polyol pathway, neuroinflammation, diacylglycerol protein kinase C (PKC) pathway activation, accumulation of advanced glycosylation end products (AGEs), and poly ADP-ribose polymerase (PARP) activity increased, Nacetyl glucosamine through homocysteine pathway enhanced protein modification and neurotrophin reduction (3, 5, 6). Many of these mechanisms are intrinsically linked. Despite various preclinical trials targeting pathological features of DPN including accumulation of AGEs, PARP, activation of PKC, activation of polyol pathway and hexosamine pathway, oxidative stress and inflammation have produced some beneficial effects in animal models, all clinical trials for modifying DPN progression have failed (6–9). DPN typically manifests as numbness at the ends of the extremities in a stocking-glove pattern, with the feet in particular being affected ealier and more severely (10). In addition the symptoms of DPN are pain, autonomic and motor neuropathy (11).

Axonal transport plays an instrumental role in neuronal development, the ability to perform normal function and post-injury regeneration. Over the past decade, the significance of axonal transport in neurological disorders has become increasingly clear. Impaired axonal transport, as an influential cause of DPN caused by or exacerbated by diabetes, is strongly linked to the onset and progression of DPN. This seems to be a common thread in most DPNs. Strengthening axonal transport is favorable to the outcome of neurological disorders (12–14). Axonal transport disorders appear early in diabetes, and abnormalities in axonal transport further promotes the pathological progression of DPN. Under normal conditions Axons receive a supply of lipids, proteins, and organelles from the soma (via retrograde transport), while components requiring degradation or recycling are transported back to the cell body (via retrograde transport). Thus structural integrity is critical for neuronal microtubules to serve as stable tracks for long-distance transport of neurofilament (NF), proteins, vesicles containing multiple neurotransmitters, organelles, and Nerve growth factor (NGF) (15, 16).Rapid retrograde axonal transport includes distal nutritional signals (such as autophagy) from the axon to somatic cell transport distal, as well as protein misfolding and aggregation (17).What this process does is return fragmented organelles and membrane constituents to the lysosome for processing and digestion, and possibly transmit information about the status of axons and nerve endings to the cell body (15, 18). In addition, mitochondria, some endosomal groups, lysosomes, and mRNAs undergo bidirectional transport (17). Axonal transport maintains a stable balance between the motor and quiescent states, thereby maintaining neuronal development, function, and survival and protecting the integrity of the entire neuron (19). Diminished anterograde transport, inability of proteins and lipids to reach distal synapses, and inability of mitochondria to meet local energy demands, lead to progressive abnormalities in peripheral sensory nerves, manifesting as hypoesthesia in a sock and glove pattern and eventually sensory loss (13, 20, 21). When axonal transport is damaged, excessive retention and accumulation will lead to mitochondrial metabolic dysfunction, diminished membrane potential, enhanced reactive oxygen species, and calcium overload, thereby damaging mitochondrial function and causing serious toxic effects on cells (22–28). Increasing the transport rate of mitochondria in damaged proximal axons can promote neural regeneration (29, 30), and retrograde transport the injured mitochondria to the cell body for repair or degradation. In streptozocin-induced diabetic rats, impairment of axonal slow transport caused by altered proximal and distal characterization of axonal caliber caused by NF. Specifically, large microtubule cytoskeletal proteins accumulate in the proximal axon region, causing axons to rise and cytoskeletal proteins reaching the distal axons cytoskeleton protein to shrink their size. This results in hypertrophy of the proximal axon area of hypertrophy and thinning of the distal part of the motor neuron dysfunction, with impaired axonal transport (31). In addition, axonal transport allows neurons to respond adequately to distal nutritional and stress signals (32). Failure of rapid axonal transport results in degeneration of nerve fibers, and the damaged nerve fibers aim to regenerate. Although they are vigorous, they are shortlived and a large number of regenerated buds cannot survive. Consequently, neuropathy steadily deteriorates (33).

Hence, any defect involving this hub could contribute to cellular dysfunction and degeneration. This article reviews the mechanisms of axonal transport and their relationship to DPN and provides an outlook on potential future therapeutic targets to enhance the understanding of DPN pathogenesis as well as to provide future research directions and possibilities.

## 2 Axonal transport

### 2.1 Axonal transport mechanism

#### 2.1.1 Cytoskeleton

**Figure 1:**
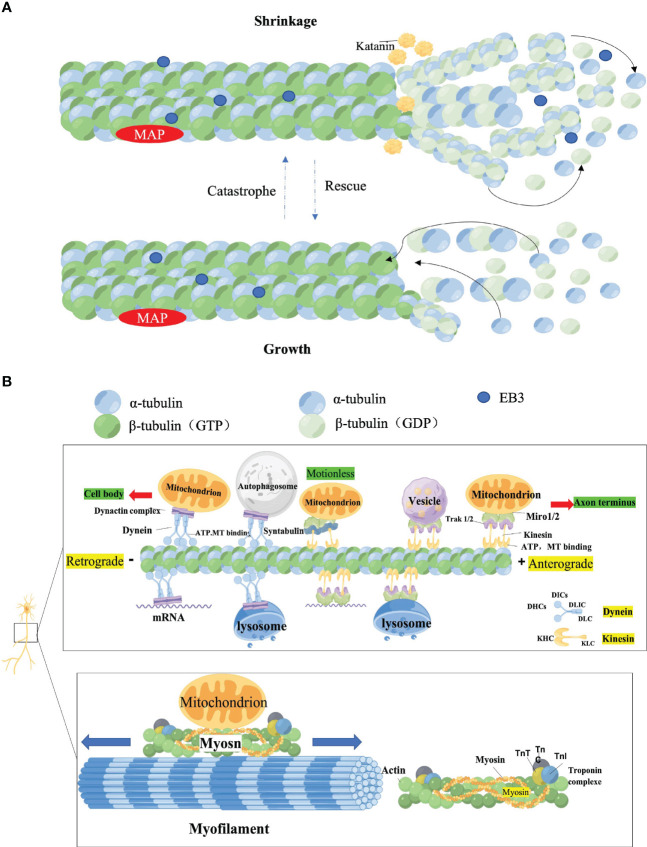
**(A)** Assembly of microtubules (MT). MTs are heterodimers of α-microtubulin and β-microtubulin, arranged radially with positive ends (positive end) facing the periphery and negative ends (–end, negative-end) facing the cytosol. The microtubule-associated protein Katanin cuts off the centrosomal MTs, releasing small and dynamic MTs of different sizes that can be delivered to axons or exons. MTs can proceed concurrent cycles of convergence (growth) and depreciation (contraction) at their plus ends. Microtubule Associated Proteins (MAPs) EB protein, a microtubule-associated protein belonging to the MT plus end tracking protein (+TIPs), binds to EB3 and accumulates at the apex when MTs grow, and dissociates when growth stops or switches from growth to contraction (MTs become smaller). EB3 is also known to regulate microtubule dynamics during axonal outgrowth. **(B)** Axonal transport. Molecular motors, dependent MT and ATP, driven the long-distance transport Microtubules bind to motor proteins, which then link cargo through adaptor proteins to form a cargo transport complex, and the motor proteolysis of ATP provides energy to ensure a smooth process. The major components of molecular motors are Kinesin, Dynein, and Myosin. Kinesin directs the plus end, and Kinesin coordinates the anterograde transport of cargo (such as Vesicle) from the cell body to the axon end along microtubules. Kinesin includes two heavy (KHC) and two light (KLC) chains. The motor domain of KHC, with ATPase activity can bind directly to MTs, whereas its C-terminal domain Interact with KLC or interact with cargo. Dynein coordinates the retrograde transport of cargo such as autophagosomes from axon terminals to the cell body along microtubules. Dynein consists of two Dynein heavy chains (DHCs) and distinct intermediates (DICs), mild intermediates (DLIC), and light chains (DLC). It is mainly required to bind Dynactin (dynein-activating protein) to form the dynein-dynactin complex to bind cargo for axonal transport. In addition, mRNA, Lysosome, and mitochondrial were transported along the axon in both directions. Mitochondria can also use myosin motors for bidirectional transport over short distances along actin filaments. Syntaphilin (SNPH) is a static anchoring protein of axon mitochondria. The MiRO1-TraK2 adaptor complex acts as an important regulator of neuronal cargo transport and increases mitochondrial motility by anchoring mitochondria to MTs.

Cargo is transported along the cytoskeleton, which includes actin filament (AF), NF, and microtubule (MT). While all cytoskeletal components are critical for the morphology and function of neurons, axonal transport is almost exclusively determined by MT and its related molecular motors: kinesin, dynein, and myosin (34, 35). MT, the primary track for intra-axonal cargo transport, is a hollow cylindrical structure composed of microtubule proteins (MTs) and microtubule-associated proteins (MAPs). Among them, the aggregation of α-microtubulin heterodimers and β-microtubulin heterodimers is the foundation for the correct orientation of axonal transport. The negative end of α-microtubulin generally is directed towards the cytosol, while the positive end of β-microtubulin faces the axon terminal (34, 35). The positive end of the MT is arranged radially toward the periphery (+ end, positive end). Katanin, a protein associated with microtubules with ATPase activity, is able to sever the central MT and release small viable MTs of varying sizes that can be delivered to axons or synapses (34, 35). Kinesin or Dynein then drives MTs located at the same polarity (negative or positive end) for transport along axons. MTs undergo both polymerization (growth) and depolymerization (shrinkage) cycles during transport, which is described as the dynamic instability of MT (36–38). The activity of intrinsic GTPases in MTs and MAPs intervenes in the transformation of MTs from growth to contraction (mutation) or from contraction to growth (rescue) (39). MAPs binds to MT full length, altering its structure, stability and kinetics. Changes in axonal cytoskeletal integrity in DPN support reduced axonal diameter, impaired axonal transport and reduced neural regeneration (39, 40) (**Figure 1**).

#### 2.1.2 Molecular motor

In the cytoplasm of neurons, cargo is attached *via* adapters to the corresponding motor proteins, which in turn are attached to the MTs. This process requires the hydrolysis of ATP by motor proteins to provide the driving force for the smooth transport of cargo along the track (41). Various major molecular motors interact to maintain normal axonal transport and are involved in different mechanisms and regulation of cargo axonal transport. In general, kinesins drive the cis-transport of cargo, i.e. the cis-transport from the cell body to the axon terminal is actuated by the motor protein superfamily of kinesins, a process that delivers RNA, proteins and organelles to the growth cones and synapses (42). In contrast, retrograde transport is Dynein-dependent, which is critical for neurotrophic factor signaling, autophagylysosome decay, and response to neural damage (42). In addition, Myosin regulates the short-distance transport of cargo-directed actin filaments (42) (**Figure 1**).

### 2.2 Impaired axonal transport promotes DPN progression

#### 2.2.1 Axonal degeneration

**Figure 2:**
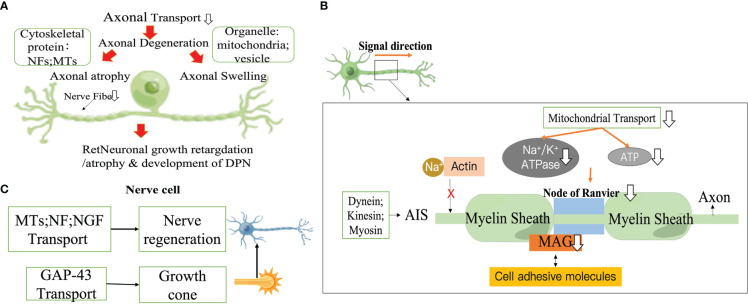
Impaired axonal transport promotes DPN progression. **(A)** Axonal degeneration. **(B)** Action potential drop. **(C)** Impaired nerve regeneration. NF, neurofilament; MT, Microtubule; MAG, Myelin associated glycoprotein; AIS, Axon initiation segment; NGF, Neurotrophic factors.

Axonal atrophy is the most common pathological feature in type 1 diabetic peripheral neuropathy, manifesting as persistent peripheral nerve fiber loss (43–45). The size of fibers is regulated by the axonal cytoskeleton, and the reduction in axon diameter is associated with a decrease in slow transport proteins (structural proteins) delivered to the axon. In particular, NF, which is the main determinant of axon size (45), directly affects the caliber of the axon, and the number of axons is closely related to the cross-sectional size of the axon with medulla (46–48). In STZ-induced the sciatic nerve (SCN) in DM rats, delayed axonal transport of proteins such as NFs and MTs is not compensated for by delayed protein synthesis, delayed half-lives, or delayed transport volume (31, 49). Furthermore, abnormally phosphorylated NFs are unable to align and interact with other cytoskeletal components, resulting in impaired axonal function and eventual atrophy and loss. This is first manifested by the loss of sensory epidermal fibers (50–53). As this death process proceeds, it causes peripheral nerve stem fibers loss, which begins with the distal nerves and progress to more proximal nerves. Axon diameter in turn affects the fundamental biological characteristics of neurofibers, such as conduction velocity (The increases conduction velocity in proportion to the square root of the interior diameter (54)), excitability and degree of myelination. Another potential pathological feature associated with DPN IENFD deficiency is axonal swelling (55–57). These axonal swellings may be associated with symptoms of sensory enhancement in patients with probable microfibrillary neuropathy (58, 59). Microstructural studies by Ebenezer and colleagues targeting the microstructure of axonal swellings in sensory neuropathies have shown that axonal swelling contains an accumulation of mitochondria, vesicular organelles and NF (60). When axonal transport is disrupted, cargo accumulates abnormally, leading to axonal swelling (58, 61). This is followed by secondary axonal detachment and Wallerian degeneration induced by the damaged axonal transport (62). Axonal degeneration is a predominant pathological change in many peripheral neuropathies, including DPN (63–67) (**Figure 2**).

#### 2.2.2 Action potential drop

Myelin, the multilaminar sheath on axons,greatly speeds neurotransmission by fundamentally changing the way action potentials are propagated.The myelin sheath that insulates the axon allows action potential to be conducted in a skipping fashion, which conducts faster and consumes less energy than unmyelinated axons. Myelinated axons of nerve cells are organized into a series of polarized fields centered on the Ranvier's node. These structural domains are multimeric protein complexes composed of different cell adhesive molecules, as well as ion channels and scaffold domains of different diameters, that are essential for normal jump conduction, axonal transport rates, organelle and skeletal component distribution (68–70). The extracellular matrix (ECM) receptors of the PNS are located in the outer membrane of axon, which is composed of dense myelin,and in the endosome of axon (especially myelin-associated glycoprotein (MAG)) that mediate interactions with axons (71, 72). A several studies have reported significant differences in the intracellular transport of freshly synthesized MAG at Ranvier's node (69). In an early experimental model of diabetes, MAG showed retrograde transport abnormalities (73), exacerbating the reduced nerve conduction velocity as well as the reduced diameter of myelinated fibers in diabetic rats (73–77). Ndel1 is a modulator of kinesin which can be stably anchored to the axon initiation segment (AIS) by interacting with Gankyrin-G and initiates the vesicular cycle through kinesin transport in the AIS (78). Structural maintenance of AIS is closely related to actin-related proteins, casein kinase2 (CK2), myosin ring-associated proteins (actin ring-associated protein, actin ring-associated protein), myosin light chain (MLC) and tropomyosin (Tpm) 3.1 (79–81). Na+ is prevented from entering the AIS after Na+ channels is tightly bound to the actin cytoskeleton (82). ATP at sites of high energy demand (e.g., active growth cones, synapses, axonal branches, or Ranffian nodes) is reduced if mitochondrial transport in axons is impaired (83–86). In the Ranvier's node, impaired mitochondrial transport impairs Na+/K+ ATPase activity, which in return promotes the reversal of axonal membrane Na+/Ca2+ transport proteins and induces an increase in cytoplasmic Ca2+ levels, thereby initiating a number of degenerative pathological processes (87, 88). Because action potentials propagate along axons, it takes a lot of energy to get neurons to transmit action potentials along the identical length of axons. In demyelinating neuropathies, enhanced transport of cargoes (e.g., mitochondria) by axons contributes to their redistribution along axons to areas where demyelination leads to increased ATP demand, which may be a useful therapeutic strategy (**Figure 2**).

#### 2.2.3 Impaired nerve regeneration

There are various potential mechanisms for the adverse effects of hyperglycemia on peripheral nervous system (PNS). Progressive neuropathy deteriorates not only by nerve fiber degeneration, but also by damaged nerve fibers attempting to regenerate, but but are short-lived despite their vigor and fail to survive even when they produce a large number of regenerative shoots. Failure of fast axonal transport causes this deterioration occurring in a distalto-proximal order (33). Regulation of MTs and NF expression and reduced synthesis/transport of neurotrophic factor have been shown to be linked to defective axonal regeneration in diabetic animals (89–91). Furthermore, aberrations in the growth cone which is the first step in neuronal regeneration can depress the success of regeneration. Growth-associated protein 43 (GAP-43) can be translocated from the cell body to the distal axon *via* rapid axonal transport within the vesicle. Basic experiments have confirmed that GAP-43 deficiency can contribute to abnormal growth cones. In ligation and extrusion experiments in STZ-induced DM rats, it was concluded that immunostaining for GAP-43 was reduced on neuronal proximal peduncles (92). Changes in axonal transport rates in diabetic patients may involve presynaptic calmodulin supply and mechanisms associated with GAP-43 disruption (93). *In vitro*, GAP -43 binds to calmodulin at moderate Ca2+ concentrations and dissociates from calmodulin at higher Ca2+ concentrations, while hyperglycemia-induced phosphorylation of protein kinase eliminates this calcium dependence (94–96) (**Figure 2**).

## 3 Mechanisms of axonal transport injury

### 3.1 Glycosylation

**Figure 3:**
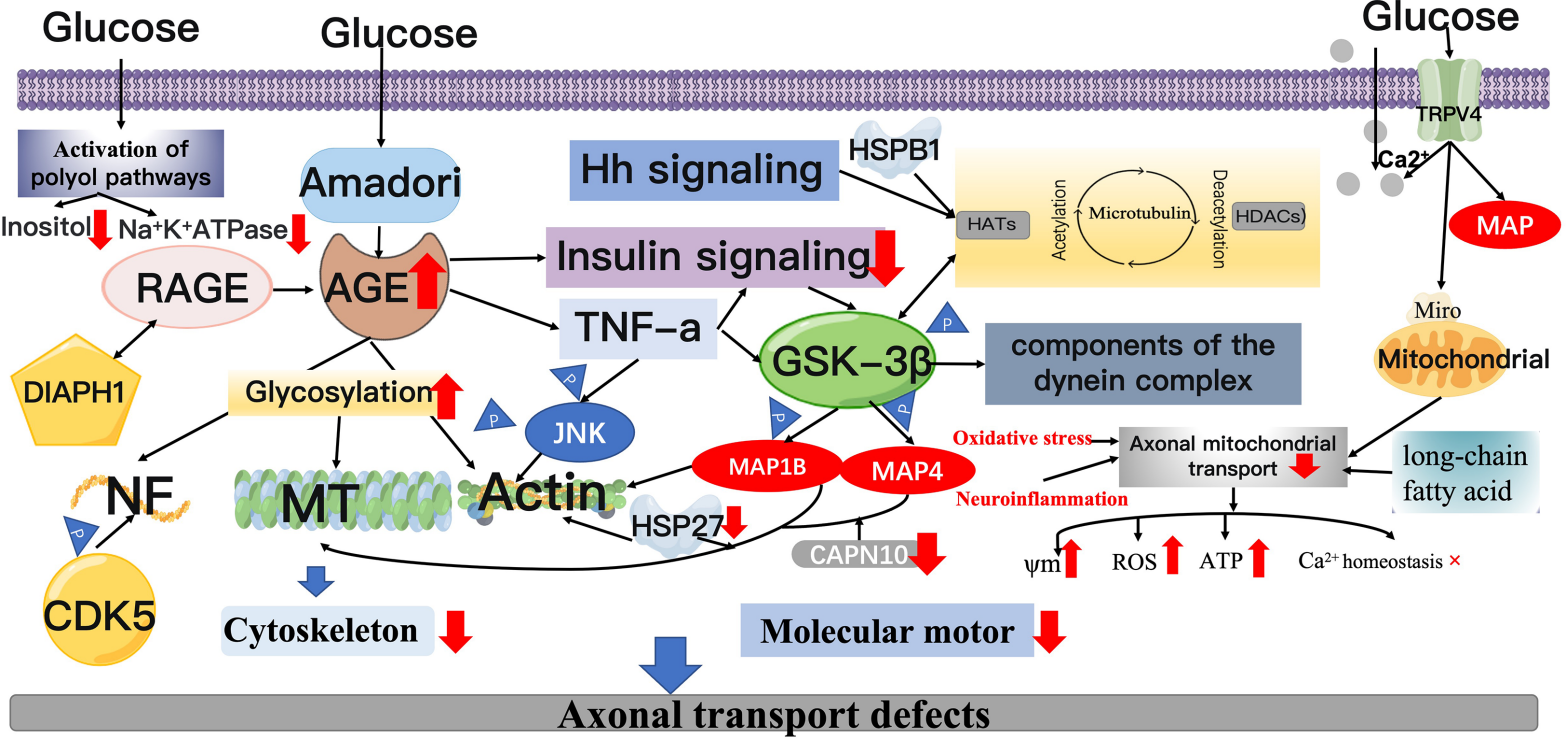
Mechanisms of axonal transport injury. AGEs: Hyperglycemia induces the production of Amadori compounds, which undergo a series of changes to form AGEs, which are deposited on the cytoskeleton of peripheral nerve cells. AGEs bind to its receptor, RAGE. On the one hand, the RAGE cytoplasmic structural domain can inhibit GTP-dependent MT aggregation by associating with DIAPH1 on the cytoskeleton, and on the other hand, it can impair axonal transport by interacting with the actin-binding protein mDia1.Espectively, regulate the acetylation and deacetylation cycle of microtubulin in MTs *via* lys40; GSK-3β can inhibit the phosphorylation of MAP1B, MAP4, and dynein, affecting MT assembly and stability and actin filament remodeling. GSK-3β can also directly phosphorylate dynein intermediate chains. The NDEL1 bound region that promotes dynein export. CDK5 can be modified by posttranslational modifications and phosphorylation of NF. JNK3 phosphorylates the motor structural domain of KIF5 and suppresses binding to MT. Heat shock proteins: Maintain the stability of actin cytoskeleton. TRPV4: TRPV4 promotes extracellular Ca^2 +^ influx, and elevated Ca^2 +^ levels may directly inhibit mitochondrial axonal transport by binding to the mitochondrial surface protein Miro. TRPV4 also contributes to MT demolition through direct binding to microtubule proteins. In combination with the direct glycosylation damage of hyperglycemia, prolonged exposure to high glucose levels induces oxidative stress, activation of polyol pathways and inflammation.

Excessive glycosylation is present in the neural tissue of diabetic patients with elevated blood glucose levels, resulting in the deposition of AGEs in various sites of diabetic peripheral nerve tissue (97). Amadori compounds appearto be the main pathway responsible for the formation of AGE products. Accumulation of Amadori glycosylation products has been demonstrated in the spinal cord of patients with amyotrophic lateral sclerosis and spinal medullary amyotrophy, possibly associated with the glycosylation of cytoskeletal proteins. Increased glycosylation of AGEs and MTs in diabetic rats can lead to abnormal axonal transport and impaired nerve growth and regeneration after 2 weeks (46, 98–100). Amino acid analysis has shown that the lysine residue was the major glycosylation site (101). This suppresses the GTP-dependent aggregation of MTs and stiffens axonal structures, thus disrupting axonal transport. However, the extent to which increased glycosylation of MTs interferes with peripheral axonal transport remains unclear. In diabetic nerves, NF protein glycosylation, NF impairment and active transport are often accompanied by histological and electrophysiological alterations, leading to long-term neurodegenerative changes (31, 46, 49, 102, 103). Binding of a receptor for advanced glycosylation end products (RAGE) with cell surface receptors and its cytoplasmic structural domain interacts with Diaphanous Related Formin 1(DIAPH1) on the cytoskeleton, whose dysfunction may lead to neuropathy. The cumulative effect of hyperglycemia on RAGE- DIAPH1-mediated signaling pathways may disrupt the organization and transport of axonal cytoskeleton (104). Axonal transport of mDia1, RAGE-interacting protein and actin binding protein was affected in RAGE knockout mice compared to wild-type mice at 3 h and 6 h after diabetic sciatic nerve injury (102). The loss of RAGE showed a positive correlated with the decrease of AGE level. The mDia1 axonal transport correlates better to diabetes-induced glycosylation of actin induced by diabetes. Glycosylated actin has been reported to be found in brain homogenates derived from diabetic animals and in platelets from early diabetic patients (102, 105). Alterations in the structure of actin may affect mDia1 actin interactions, leading to impaired translocation between the two. In addition, research on extracellular matrix proteins found in diabetic nerve endosomes suggests that premature glycosylation of these proteins affects axon growth (102, 106). Osonoi et al. reported that high glucose induced the activation of AGE/RAGE signaling pathway, which could further promote the progress of DPN by reducing insulin signaling pathway and inducing macrophage activation. Phosphorylation of TNF-α directly stimulates JNK, thereby disrupting retrograde axonal transport. On the other hand, TNF-α stimulation attenuates insulin signaling in neuronal cells. GSK3β is located downstream of insulin and JNK signaling, which phosphorylates components of the dynein complex and decreases retrograde vesicle transport (107) (**Figure 3**).

Current evidence suggests that the fast axonal transport rates may not be affected by RAGE (102). This is because glycosylation of proteins associated with diabetes is a relatively long process and the rapid transport of axons is so fast that hyperglycemia does not to mediate this change. STZ-induced DM rats have highly increased microtubule protein glycosylation in peripheral nerves, but brain microtubule protein glycosylation in controls did not show any increased changes (102), suggesting that inhibition of microtubule assembly in the brain is independent of the the level of glycosylation (106). The mechanisms of impaired axonal transport are very complex in nature and are not limited to a single cause (108).

### 3.2 Post-translational modification

The main modality of MT regulation is *via* post-translational modifications (PTMs), involving acetylation, phosphorylation and glutamylation (109–111). Microtubule protein PTMs abnormalities in sensory neurons have been related to axonal regeneration damage (112, 113).

Acetylation modifications of microtubule protein can promote self-healing and stabilization of MT (114–117). The α/β heterodimeric a-subunit of Lys40 in MTs is the site of acetylation modification of microtubulin (114), which can determine MT motor processivity (118–121). Kinesin-1 reacts preferentially with acetylated and demethylated MT (120, 122, 123). In fact, acetylation-modified MTs is the preferred pathway for driving protein 1-independent mitochondrial translocation, and the ER/mitochondrial contact and mitochondrial fusion/division also selectively take place on acetylated MTs (117, 124–126). Histone acetyltransferases (HATs) and histone deacetylases (HDACs) are involved in the acetylation modification of microtubulin, as well as their regulation of the acetylation and deacetylation loops of lys40 on MTs in -microtubulin, respectively (117). Histone deacetylase 6 (HDAC 6) is a cytoplasmic class II histone deacetylase that targets acetyl groups on posttranslationally modified non-histone proteins (e.g., microtubules) to with significant alteration the target protein (127). Microtubule stability is affected by the balance between HDAC6 and HAT1, and once this balance is disrupted, cellular damage is induced. In particular, increased HDAC6 activity is specifically detrimental to neurons (128, 129), and its dysregulation is closely interlinked with the occurrence of peripheral neuropathy, neuronal microtubule instability, and reduced mitochondrial axonal transport (130–132).

Serine protein kinase 3β (GSK-3β), cyclin-dependent kinase 5 (CDK5), and c-jun NH2 -terminal kinase 3 (JNK3)have affinity for cytoskeletal elements (133–135). GSK-3β is a potent protein phosphorylation stress kinase. A whole transcriptomic study of SCN in experimental models of T1DM and T2DM found that downregulation of microtubule-associated protein 1B (MAP1B) and microtubuleassociated protein 4 (MAP4) was associated with common alterations in the structure of SCNs (136, 137). It is hypothesized that this is associated with impaired insulin and IGF-1 signaling pathways, leading to reduced GSK-3β, which inhibits phosphorylation of MAP1B. MAP1B coordinates the remodeling of MT and actin filaments within neuronal cells, regulates MT assembly and stability (138, 139), provides the basis for axonal transport and polarity, and plays an essential part in the evolution and sustenance of the PNS network (140). GSK-3β directly phosphorylates MAP and kinesin in neurons, and the binding of MAP and MT is enhanced, further regulating MT kinetics (141). Furthermore, MAP1B is the first candidate susceptibility gene for type 2 diabetes (138). Calpain-10 (CAPN10) is a part of the calpain family of enzymes, which coordinates its binding activity to MT and actin filaments by processing microtubule-associated protein 1 (MAP1) family proteins to form heavy and light chains (142). Impaired MT-F actin integration and abnormal actin dynamics are associated with deletion of CAPN10 and MAP1B. It was found that insulin and glucagon secretion was significantly increased in CAPN10 knockout mice (142) (**Figure 3**).

In addition, GSK-3β negatively modulates the kinesin engagement with NDEL1. GSK -3β directly phosphorylates the intermediate chain of kinesin, which is a key site in the binding region of the protein to NDEL1, and can facilitate kinesin output. Enhancement of insulin signal or direct GSK-3β suppression can activate kinesin movement (143). The reduction of GSK-3β activity enhances transport capacity and leads to retrograde migration of organelles compared to quiescent organelles, such as eosinophilic organelles, but this has little effect on anterograde transport. In most cases, phosphorylation coordinates their binding to MTS, which may involve the movement of dynamic proteins in many ways. TRAK1 is the key protein that connects the MOM protein Miro to the motor kinesin and kinetic protein of the molecule (144). GSK-3β has been described to be essential for the function of kinesin and dynein proteins (145). GSK-3β forms a complex by physically binding to TRAK1, DISC1 and NDE1, though GSK-3β does not alter mitochondrial motility speed or mitochondrial excursion length (146). The Hedgehog (Hh) signaling pathway can affect the level of MT acetylation in mammalian cells. Activity of the Hh pathway induces an increase in the level of MT-associated DYRKiB kinase, inhibits GSK3 β *via* phosphorylation of serine 9 and subsequently inhibits HDAC6 enzyme activity, which in turn promotes acetylation levels of MT. The Hh signaling pathway can promote MT non-independent processing by activating of DRKiB, such as intracellular mitochondrial transport, polarization of mesenchymal cells and directed cell migration (**Figure 3**).

Phosphorylation of NFs is influenced by CDK5 expression (46, 147), and abnormalities in this process are present in peripheral nerve injury (49, 98, 148, 149). Aberrant phosphorylation of neurofilament proteins (NFs) and abnormal MTs caused by NF-related protein kinases were found in dorsal root ganglion cells (DRGs) of DPN rats, a result that led to reduced axonal transport of NFs and progressive defects in axonal function (147, 150, 151). NF mRNA is not elevated during development and radial growth, therefore post-transcriptional regulation of NF appears to be more important. The stability of NF transcript is mediated by various neurotrophic molecules like nerve growth factor (NGF), NT-3, IGF-1, insulin and c-peptide.

Studies have revealed that in the SCN of experimental DPN rats, retrograde transport of NGF is impaired and abnormal phosphorylation of NF disrupts its arrangement and interaction with other cytoskeletal components, leading to compromised axonal functions and eventual atrophy and loss (152). In addition to MT changes, insufficient or incorrect NF protein synthesis can also severely damage the axonal cytoskeleton, and abnormal NF expression, processing, and structure can lead to DPN progression (153) (**Figure 3**).

JNK3 phosphorylates the KIF5 locomotor region and suppresses its binding to MT (154, 155). JNK, p38, and ERK are activated in DRG and in the sural nerve with the potential to mediate neurodegenerative disseases (156, 157). This may be connected to the fact that activation of JNK and ERK mediates abnormal phosphorylation of NF in DM sensory neurons, leading to axon diameter loss and nerve terminal death (157). However, the enhanced DM-induced activation of JNK and p38 is limited to the anterograde portion of axonal transport (157).

### 3.3 Heat shock proteins

Heat shock proteins (HSPs) are a class of highly conserved proteins discovered in 1962. HSPs, have been demonstrated to be upregulated in many neurological diseases as a mechanism to counteract the aggregation or formation of abnormal proteins identified in disease conditions (158, 159). Minor heat shock protein B1(HSPB1, also referred to as HSP27) is broadly expressed *in vivo* (160). HSP27 is associated with neuronal survival and hereditary neuropathy, which is reported to be essential for the recovery of both sensory and motor neurons (159, 161–163). Overexpression of HSP27 occurs in some target tissues of diabetes complications (163–166). In mice, knockdown or overexpression of HSP27 correlates with diminished or enhanced regenerative properties after nerve injury (167–169). The neuroprotective mechanism may be related to the stabilization of the actin cytoskeleton (170). Overexpression of transgene HSP27 in T1DM mice displays protective effects against loss of thermal sensation, mechanical nociceptive sensitization, epidermal innervation loss and delayed sensory transmission (171). Serum levels of HSP27 (sHSP27) might thus be a novel biomarker of DPN (172): sHSP27 expression levels in T1DM are independently associated with distal symmetric polyneuropathy, and low serum levels of HSP27 are linked to extensive nerve fiber dysfunction (173). In the peripheral nerve samples from asymptomatic mutant HSPB1 transgenic mice, it was discovered that it did not modify the level of acetylated α-tubulin, so its stabilizing effect on microtubules was enhanced (174). Interestingly, in symptomatic mutant HSPB1 transgenic mice, acetylated α-tubulin levels were decreased and microtubule instability was raised, possibly due to enhanced HDAC6 recruitment (174). Defective axonal transport is related to the downregulation of acetylated α-microtubulin, and this instability can result in defective axonal transport (175). This transport defect can be remedied by the use of the optional HDAC6 inhibitor Tubastatin A or the class I and class II HDAC inhibitors trigonelline A (176). Similarly, Kim et al. recently found defective mitochondrial axonal transport in motor interneurons extracted in patients carrying the HSPB1 synapse, and this could also be repaired by using specific HDAC6 inhibitors (177). HSPB1 protects well against diabetic distal polyneuropathy in DM mice, as overexpression of HSPB1 in neurons of DM mice can protect against a range of neuropathies, including mechanical hyperalgesia, loss of footpad thermal sensation, reduced sensory conduction velocity, and loss of epidermal innervation (178). The function of P150 provides the link between various cellular cargoes and the reverse molecule kinesin, as well as governing kinesin movement (179) Mutant HSPB1 colocalizes with P150, causing mislocalization of P150 in the cell, impeding retrograde transport necessary for cell function and survival, specifically for motor neurons. Nonetheless, mitochondrial transport defects were not involved, possibly because the HSPB1 mutation has no effect on anterograde axonal transport, or perhaps only some anterograde transport substances are disrupted (180) (**Figure 3**).

### 3.4 Molecular motor

Forty-five kinesin motor genes have been identified, among which the kinesin-1 family (KIF5) is the core motor gene responsible for driving neuronal cargo transport (155, 181). KIF5A, KIF5B and KIF5C in mammalian KIF5 genes are all expressed in neurons, but at different levels in different cell types (182, 183). In STZ-induced DM rats, KIF5B levels are elevated in the SCN and KIF5B mRNA expression was increased in the spinal sensory and motor neurons (184). Recently, various studies have indicated that expression is low in the dorsal root ganglion (DRG) neurons of diabetic male rats (185). Upregulation of KIF5B expression may be a compensatory mechanism associated with the re-establishment of axonal motor levels. KIF5A is involved in both fast axonal transport in mitochondria and slow axonal transport in NFs. NFs are involved from the early stages of the disease and the accumulation of these NFs in somatic cells leads to the depletion, loss and degeneration of large diameter axons (186–190). KIF5A translocation plays an essential role in PI3Kmediated cell survival and sensitization of supporting neurons (191). KIF1A participates in the trafficking of synaptic vesicles, NGF receptors, and TrkA (192), and any changes in this kinesin may promote the progression of diabetic neuropathy (191). Children with KIF1A mutations exhibit global developmental delay, mental retardation, bilateral lower extremity weakness, and diabetes mellitus (193).

Interestingly, there are gender differences in the expression of axonal motor proteins in DM (194), and Pesares et al. observed in early DM that the the mRNA level protein content of kinesin family members KIF1A, KIF5B, KIF5A and Myosin was altered only in male rats (185). Both the time to onset, incidence and severity of neuropathy are higher in males than in females (195–197). The decline in IENFD is more pronounced in males presenting with neuropath, such as high contraction thresholds in the foot and paw (198, 199). It is speculated that it may be related to neuroactive steroid activity. Neuroactive steroids are able to regulate mitochondrial function, which plays an essential part in axonal transport through the production of ATP (the energy source for movement). The gender-specific alterations in this motor protein identified in animal models of diabetes may eventually explain the gender differences in pain and analgesia observed in DPN (197, 198). Furthermore, expression of activated dynamin-1-like protein (DRP1) is significantly enhanced in the DRG of male DM patients, with rapid mitosis ultimately leading to unhealthy mitochondria (20). It promotes mitochondrial fragmentation and may impair mitochondrial function. Reduced transit ATP content further exacerbates axonal transit damage.

### 3.5 TRPV4 ion channel

Transient receptor potential cation channel subfamily V member 4 (TRPV4) is located in the cell membrane of sensory cell neurons derived from the PNS and other cell types throughout the body. Upon activation, TRPV4 promotes extracellular Ca2+ influx. In the adipose tissue of prediabetic mice fed with high-fat diet, high expression of TRPV4 leads to an injurious hypersensitivity response (200). Ca2+ coordinates the start of fast axonal transport as well as the stable transport of intraaxonal cargo (201, 202). Ca2+ fluctuations gravely alter mitochondrial function and movement (203–205). For example, rising Ca2+ levels can inhibit mitochondrial axonal transport directly through Ca2+ association with the Miro, a mitochondrial surfactant protein involved in docking with the motor domain of kinesin-1 (206). Moreover, the interaction of TRPV4 with MAP Ensconsin is an essential requirement for kinesin-1 (207–209).

Treatment of STZ-induced DM mice with the selective TRPV4 channel antagonist HC067047 markedly inhibited mechanical hyperalgesia (210, 211). Ca2+ influx in neurons occurs in the presynaptic-post-synaptic membrane, where a large amount of energy is required to maintain ionic gradients and mitochondrial abundance. In addition, when local ATP levels are relatively low, the ability of Ca2+ to pump across the cytoplasmic membrane is compromised, resulting in a sustained rise in Ca2+ in the cytoplasm. Therefore, locally elevated Ca2+ can be anchored to high energy demand and low supply by blocking the passing mitochondria. Conversely, where ATP is high with excess mitochondria, Ca2+ will be low and mitochondria will migrate freely.TRPV4 can also bind directly to microtubule proteins, leading to MT disassembly (212) (**Figure 3**).

### 3.6 Other

In parallel to direct glycosylation damage caused by hyperglycemia, chronic exposure to high levels of glucose can induce oxidative stress, activation of polyol pathways, and inflammation. All types of damage may interfere with axonal transport. The state of oxidative stress raises the amount of the retrograde mitochondria, while neuroinflammation increases the quantity of resting mitochondria and inhibits retrograde mitochondrial transport. Slowness of mitochondrial transport promotes an increase in ROS related to mitochondrial dysfunction, and the accumulation of mitochondrial mutations (213). In contrast, it has been indicated that anterograde transport of nerve cells exposed to ROS is more likely to be inhibited than retrograde transport (214). Increased glucose levels in neuronal cells lead to saturation of the normal glucose metabolic pathway, with excess glucose being shunted to polyol pathway and converted to sorbitol and fructose *via* the enzymes aldose reductase and sorbitol dehydrogenase (215). The accumulation of these substances causes a decrease in inositol, diminished membrane Na+/K+ ATPase activity, impaired axonal transport and disruption of neural structures, which induces the propagation of abnormal potentials (216). Treatment with an aldose reductase inhibitor reduces susceptibility to rapid axonal transport after STZ-induced nerve entrapment in diabetic rats (217, 218), and reduces the inhibition of fast axonal transport. Cis-axonal transport of choline acetyltransferase has been reported to be restored following treatment (219, 220) (**Figure 3**).

Plasma free saturated fatty acid (SFA) levels are generally higher in patients with T2DM and may be involved in the occurrence and progress of peripheral neuropathy (221). It was found that DRG neurons exposed to elevated SFAs have reduced numbers of mitochondria in their axons (222, 223). long-chain fatty acids (e.g. palmitic and stearic acids) in SFA can damage DRG neurons, and their elevated concentrations disrupt mitochondrial transport, alter mitochondrial bioenergetics (224, 225) (222), reduce the amount and speed of mitochondrial motility, and depolarize mitochondria (223). Increased levels of complex lipids such as palmitic and stearic acids in the SCN of HFD and HFD- STZ mice have been shown to impede mitochondrial function and its transport, inducing apoptosis in DRG neurons (226, 227). Increased intake of a diet rich in monounsaturated fatty acid (MUFA) reversed neuropathy and restored nerve transmission velocity and nerve fiber density within the epidermis (228), which may be related to the prevention of impaired mitochondrial transport in SFA palmitate-treated sensory neurons cultured *in vitro* by MUFA oleic acid (**Figure 3**).

In addition, axons may be affected by microvascular alterations in the vascular plexus of peripheral nerves (229) in advanced cases of diabetes. Alterations in microvascular structure, together with neurological and biochemical disorders, may lead to a decline in internal blood flow and partial oxygen pressure. Regional compression of normal peripheral nerves by 30 mmHg can cause changes in internal circulation and increased internal vascular permeability (230, 231), which may be more pronounced in DM patients. As a result of such changes, axonal transport induced by compression may be remarkably inhibited (232) (**Figure 3**).

## 4 Conclusions and future directions

Treatment for reversing impaired axonal transport is still largely in experimental animal models of DPN without effective methods to detect axonal transport in the clinic. For example, in diabetic rats, impaired cis-axonal transport can be improved by aldose reductase inhibitors or oral inositol. The addition of an aldose reductase inhibitor 3 weeks after induction of diabetes reversed defects in choline acetyltransferase axonal transport and motor nerve conduction velocity (220, 233–235), which may be related to the promotion of actin slow component B (SCb) and βtubulin (SCb) in DM rats (236). Ginkgo biloba extract (GBe), increasing the mean axonal diameter, slowed down the slow axonal transport block induced by high glucose (234). Exercise, as a non-drug pathway, exerts a beneficial effect in the treatment of DPN axonal transport defects. Endurance exercise inhibits the increase of KIF5, KIF1B, Dynein and other molecular motor protein contents in SCN of DPN rats (237), promotes axonal transport, and ameliorates nerve damage (238, 239). Diabetic rats treated with gangliosides have been proven to prevent the progression of MNCV deficiency (240–243), and another study showed that gangliosides protect against impaired axonal transport of different molecular versions of acetylcholinesterase in diabetic animals (242). In *in vitro* cultured SFA palmitatetreated sensory neurons, increased intake of a diet rich in monounsaturated fatty acid (MUFA) prevented impaired mitochondrial transport and reversed neuropathy (228). Aminoguanidine protects the cytoskeleton of DPN rats by inhibiting the accumulation of AGEs and glycosylation of structural proteins (49, 74, 149, 244–247). In cultured adult DRG neurons, Hsp27 expression promotes axonal growth (248), which may be related to its ability to promote actin polymerization (249, 250), a key component of axonal extension (251). Recent work has shown that HDAC6 inhibitors, such as Ricolinostat, an inhibitor of histone deacetylase 6, have been effective in improving DPN (252), chemotherapy-induced peripheral neuropathy (CIPN) (253, 254),and peroneal muscular dystrophy (CMT) type 2 disease (255) in animal models with good safety and tolerability. The mechanism is to inhibit HDAC6 to improve acetylation of microtubulin, promote mitochondrial translocate to the outer end of neurons, provide and maintain necessary energy and nutrition for nerve fibers, stimulate regeneration of intraepidermal nerve fibers (IENFs), restore nerve fiber function, and essentially alleviate and heal peripheral nerve injury (**Table 1**).

**Table 1:** Potential therapeutic approaches to improve axonal transport mechanisms in diabetic peripheral neuropathy.

Treatment	Pathological Changes	Mechanism	Evidence-based
Essential fatty acid (228)	Nerve conduction velocities	Mitochondrial transport Intraepidermal nerve fiber density	Animal studies Cell culture experiments
Aldose reductase inhibitors (220, 233–236); Inositol (233)	Nervous conduction velocity; Axonal transport of choline acetyltransferase	Promotion of actin slow component B (SCb) and βtubulin (SCb)	Animal studies
Ginkgo biloba extract (GBe) (234)	Disturbed slow axonal transport	Increased the mean diameter of axons	Animal studies
Exercise (237–239)	Retrograde axonal transport is impaired	Inhibit the increase in the content of molecular motor proteins	Animal studies
Gangliosides (240–243)	Prevents defects in the accumulation of systolic PFK activity; reverses damaged transport of different forms of the molecule acetylcholinesterase by axons.	Inhibit the structural breakdown of the axonal endoskeleton	Animal studies
Aminoguanidine (49, 74, 149, 244–246)	Nervous conduction velocity; Improved myelin fiber size reduced axonal atrophy	Inhibition of structural protein glycosylation, thereby preserving cytoskeletal organization.	Animal studies
Microtubule stabilizer (HDAC6 inhibitors) (252–255)	Stimulate the regeneration of intraepidermal nerve fibers, recover nerve fiber function	Increasing the acetylation of tubulin by inhibiting HDAC6	Animal studies Cell culture experiments
Hsp27 (248–251)	Enhances neurite growth	Promoting actin polymerization	Cell culture experiments

In experimental models and animal nerve samples from DPN patients, neuroanatomical and electrophysiological aberrations linked to axonal transport include reduced fiber diameter, reduced motoneuron conduction velocity, segmental demyelination and axonal lost, ultimately leading to impaired neuronal degeneration and regeneration. The metabolic mechanism of hyperglycaemia are well studied and have been recognised as impairing axonal transport and promoting the occurrence and development of DPN by activating glycosylation, polyols, oxidative stress, and inflammation. Hyperlipidemia is more extreme, and light loss due to excess saturated fatty acids contributes to the pathogenic mechanism. The insulin signaling pathway aggravates the progression of DPN through protein phosphorylation kinases and kinase phosphorylation modifying microtubules and molecular motors. In the future, the heat shock protein sHSP27 may be a novel biomarker for diabetic neuropathy by promoting microtubule stabilization to enhance axonal transport disorders and successfully restore nerve regeneration.

Even with strict glycemic control or pancreatic transplantation, established neuropathy is difficult to reverse. Hence, targeted approaches that use axonal regeneration to reverse the abnormalities are imperative. Reconstructing the axonal transport function of nerve cells is promising as a potential therapeutic target for DPN.

## Author contributions

CY conceived and wrote the article. FL proofread the data and provided guidance for writing and submission. XZ revised the grammar of the text. All authors contributed to the article and approved the submitted version.

## References

[1] Federation ID. IDF diabetes atlas 8th edition[J], International diabetes federation. (2017) 905-911.

[2] Albers JW Pop-Busui R. Diabetic neuropathy: mechanisms, emerging treatments, and subtypes. Curr Neurol Neurosci Rep (2014) 14(8):1–11. doi: 10.1007/s11910-014-0473-5 PMC5084622 24954624

[3] Singh R Kishore L Kaur N. Diabetic peripheral neuropathy: current perspective and future directions. Pharmacol Res (2014) 80:21–35. doi: 10.1016/j.phrs.2013.12.005 24373831

[4] Boulton AJ. Management of diabetic peripheral neuropathy. Clin Diabetes (2005) 23(1):9–15. doi: 10.2337/diaclin.23.1.9

[5] Farmer KL Li C Dobrowsky RT. Diabetic peripheral neuropathy: should a chaperone accompany our therapeutic approach? Pharmacol Rev (2012) 64(4):880–900. doi: 10.1124/pr.111.005314 22885705 PMC3462992

[6] Feldman EL Nave K-A Jensen TS Bennett DL. New horizons in diabetic neuropathy: mechanisms, bioenergetics, and pain. Neuron (2017) 93(6):1296–313. doi: 10.1016/j.neuron.2017.02.005 PMC5400015 28334605

[7] Obrosova IG Huysen CV Fathallah L Cao X Greene DA Stevens MJ. An aldose reductase inhibitor reverses early diabetes-induced changes in peripheral nerve function, metabolism, and antioxidative defense. FASEB J (2002) 16(1):1–26. doi: 10.1096/fj.01-0603fje 11709499

[8] Cameron N Cotter M Basso M Hohman T. Comparison of the effects of inhibitors of aldose reductase and sorbitol dehydrogenase on neurovascular function, nerve conduction and tissue polyol pathway metabolites in streptozotocin-diabetic rats. Diabetologia (1997) 40(3):271–81. doi: 10.1007/s001250050674 9084964

[9] Pop-Busui R Boulton AJ Feldman EL Bril V Freeman R Malik RA. Diabetic neuropathy: a position statement by the American diabetes association. Diabetes Care (2017) 40(1):136–54. doi: 10.2337/dc16-2042 PMC6977405 27999003

[10] Charnogursky G Lee H Lopez N. Diabetic neuropathy. Handb Clin Neurol (2014) 120:773–85. doi: 10.1016/B978-0-7020-4087-0.00051-6 24365351

[11] Tracy JA Dyck PJB. The spectrum of diabetic neuropathies. Phys Med Rehabil Clinics North America (2008) 19(1):1–26. doi: 10.1016/j.pmr.2007.10.010 PMC2720624 18194747

[12] De Vos KJ Grierson AJ Ackerley S Miller CC. Role of axonal transport in neurodegenerative diseases. Annu Rev Neurosci (2008) 31:151–73. doi: 10.1146/annurev.neuro.31.061307.090711 18558852

[13] Millecamps S Julien J-P. Axonal transport deficits and neurodegenerative diseases. Nat Rev Neurosci (2013) 14(3):161–76. doi: 10.1038/nrn3380 23361386

[14] Hinckelmann M-V Zala D Saudou F. Releasing the brake: restoring fast axonal transport in neurodegenerative disorders. Trends Cell Biol (2013) 23(12):634–43. doi: 10.1016/j.tcb.2013.08.007 24091156

[15] Brimijoin W. Abnormalities of axonal transport: are they a cause of peripheral nerve disease? Mayo Clinic Proc (1982) 57(11):707–14. doi: 10.1016/S0140-6736(82)91219-3 6182427

[16] Guillaud L El-Agamy SE Otsuki M Terenzio M. Anterograde axonal transport in neuronal homeostasis and disease. Front Mol Neurosci (2020) 13:556175. doi: 10.3389/fnmol.2020.556175 33071754 PMC7531239

[17] Olenick MA Holzbaur EL. Dynein activators and adaptors at a glance. J Cell Sci (2019) 132(6):jcs227132. doi: 10.1242/jcs.227132 30877148 PMC6451413

[18] Sidenius P Jakobsen J. Retrograde axonal transport. Diabetologia (1981) 20(2):110–2. doi: 10.1007/BF00262011 6162698

[19] Kristensson K Olsson Y. Diffusion pathways and retrograde axonal transport of protein tracers in peripheral nerves. Prog Neurobiol (1973) 1:85–109. doi: 10.1016/0301-0082(73)90017-8 4130375

[20] Vincent AM Edwards JL McLean LL Hong Y Cerri F Lopez I. Mitochondrial biogenesis and fission in axons in cell culture and animal models of diabetic neuropathy. Acta Neuropathol (2010) 120(4):477–89. doi: 10.1007/s00401-010-0697-7 PMC4254759 20473509

[21] Sajic M. Mitochondrial dynamics in peripheral neuropathies. Antioxid Redox Signaling (2014) 21(4):601–20. doi: 10.1089/ars.2013.5822 24386984

[22] Court FA Coleman MP. Mitochondria as a central sensor for axonal degenerative stimuli. Trends Neurosci (2012) 35(6):364–72. doi: 10.1016/j.tins.2012.04.001 22578891

[23] Lee CW Peng HB. The function of mitochondria in presynaptic development at the neuromuscular junction. Mol Biol Cell (2008) 19(1):150–8. doi: 10.1091/mbc.e07-05-0515 PMC2174173 17942598

[24] Attwell D Laughlin SB. An energy budget for signaling in the grey matter of the brain. J Cereb Blood Flow Metab (2001) 21(10):1133–45. doi: 10.1097/00004647-200110000-00001 11598490

[25] Verstreken P Ly CV Venken KJ Koh T-W Zhou Y Bellen HJ. Synaptic mitochondria are critical for mobilization of reserve pool vesicles at drosophila neuromuscular junctions. Neuron (2005) 47(3):365–78. doi: 10.1016/j.neuron.2005.06.018 16055061

[26] Courchet J Lewis TL Jr. Lee S Courchet V Liou D-Y Aizawa S. Terminal axon branching is regulated by the LKB1-NUAK1 kinase pathway *via* presynaptic mitochondrial capture. Cell (2013) 153(7):1510–25. doi: 10.1016/j.cell.2013.05.021 PMC3729210 23791179

[27] Spillane M Ketschek A Merianda TT Twiss JL Gallo G. Mitochondria coordinate sites of axon branching through localized intra-axonal protein synthesis. Cell Rep (2013) 5(6):1564–75. doi: 10.1016/j.celrep.2013.11.022 PMC3947524 24332852

[28] Sun T Qiao H Pan P-Y Chen Y Sheng Z-H. Motile axonal mitochondria contribute to the variability of presynaptic strength. Cell Rep (2013) 4(3):413–9. doi: 10.1016/j.celrep.2013.06.040 PMC3757511 23891000

[29] Misgeld T Kerschensteiner M Bareyre FM Burgess RW Lichtman JW. Imaging axonal transport of mitochondria *in vivo* . Nat Methods (2007) 4(7):559–61. doi: 10.1038/nmeth1055 17558414

[30] Kiryu-Seo S Tamada H Kato Y Yasuda K Ishihara N Nomura M. Mitochondrial fission is an acute and adaptive response in injured motor neurons. Sci Rep (2016) 6(1):1–14. doi: 10.1038/srep28331 27319806 PMC4913268

[31] Medori R Autilio-Gambetti L Jenich H Gambetti P. Changes in axon size and slow axonal transport are related in experimental diabetic neuropathy. Neurology (1988) 38(4):597597. doi: 10.1212/WNL.38.4.597 2451191

[32] Perlson E Maday S Fu M-M Moughamian AJ Holzbaur EL. Retrograde axonal transport: pathways to cell death? Trends Neurosci (2010) 33(7):335–44. doi: 10.1016/j.tins.2010.03.006 PMC2902719 20434225

[33] King R. The role of glycation in the pathogenesis of diabetic polyneuropathy. Mol Pathol (2001) 54(6):400. 11724915 PMC1187130

[34] Hartman JJ Vale RD. Microtubule disassembly by ATP-dependent oligomerization of the AAA enzyme katanin. Science (1999) 286(5440):782–5. doi: 10.1126/science.286.5440.782 10531065

[35] Rezabkova L Jiang K Capitani G Prota AE Akhmanova A Steinmetz MO. Structural basis of katanin p60: p80 complex formation. Sci Rep (2017) 7(1):1–8. doi: 10.1038/s41598-017-14194-2 29097679 PMC5668312

[36] Horio T Murata T. The role of dynamic instability in microtubule organization. Front Plant Sci (2014) 5:511. doi: 10.3389/fpls.2014.00511 25339962 PMC4188131

[37] Weisenberg RC. Microtubule formation *in vitro* in solutions containing low calcium concentrations. Science (1972) 177(4054):1104–5. doi: 10.1126/science.177.4054.1104 4626639

[38] Hines TJ Smith DS Twiss JL Roossien DH Kalinski AL. Cell communication: Prototypic integrative processes-neuronal transport and spatial signaling mechanisms in neural repair. (2022). doi: 10.1016/B978-0-12-821618-7.00161-9

[39] Shelanski ML Gaskin F Cantor CR. Microtubule assembly in the absence of added nucleotides. Proc Natl Acad Sci (1973) 70(3):765–8. doi: 10.1073/pnas.70.3.765 PMC433354 4514990

[40] Macioce P Filliatreau G Figliomeni B Hassig R Thiéry J Giamberardin LD. Slow axonal transport impairment of cytoskeletal proteins in streptozociti-induced diabetic neuropathy. J Neurochem (1989) 53(4):1261–7. doi: 10.1111/j.1471-4159.1989.tb07423.x 2475585

[41] Maday S Twelvetrees AE Moughamian AJ Holzbaur EL. Axonal transport: cargo-specific mechanisms of motility and regulation. Neuron (2014) 84(2):292–309. doi: 10.1016/j.neuron.2014.10.019 25374356 PMC4269290

[42] Hammer JA Wagner W. Functions of class V myosins in neurons. J Biol Chem (2013) 288(40):28428–34. doi: 10.1074/jbc.R113.514497 PMC3789944 23990471

[43] Sugimoto K Murakawa Y Sima A. Diabetic neuropathy–a continuing enigma. Diabetes/Metab Res Rev (2000) 16(6):408–33. doi: 10.1002/1520-7560(200011/12)16:6<408::AID-DMRR158>3.0.CO;2-R 11114101

[44] Greene DA Sima AA Stevens MJ Feldman EL Lattimer SA. Complications: neuropathy, pathogenetic considerations. Diabetes Care (1992) 15(12):1902–25. doi: 10.2337/diacare.15.12.1902 1464245

[45] Sima A Zhang W Sugimoto K Henry D Li Z Wahren J. C-peptide prevents and improves chronic type I diabetic polyneuropathy in the BB/Wor rat. Diabetologia (2001) 44(7):889–97. doi: 10.1007/s001250100570 11508275

[46] McLean W. The role of the axonal cytoskeleton in diabetic neuropathy. Neurochem Res (1997) 22(8):951–6. doi: 10.1023/A:1022466624223 9239750

[47] Griffin JW Anthony DC Fahnestock KE Hoffman PN Graham DG. 3, 4-Dimethyl-2, 5hexanedione impairs the axonal transport of neurofilament proteins. J Neurosci (1984) 4(6):1516–26. doi: 10.1523/JNEUROSCI.04-06-01516.1984 PMC6564981 6202854

[48] Griffin J Cork LC Troncoso JC Price DL. Experimental neurotoxic disorders of motor neurons: neurofibrillary pathology. Adv Neurol (1982) 36:419–33. doi: 10.1097/01.jnen.0000268833.00285.6c 6891174

[49] Medori R Autilio-Gambetti L Monaco S Gambetti P. Experimental diabetic neuropathy: impairment of slow transport with changes in axon cross-sectional area. Proc Natl Acad Sci (1985) 82(22):7716–20. doi: 10.1073/pnas.82.22.7716 PMC391404 2415969

[50] Brismar T Sima AA. Changes in nodal function in nerve fibres of the spontaneously diabetic BB-wistar rat: potential clamp analysis. Acta Physiol Scand (1981) 113(4):499–506. doi: 10.1111/j.1748-1716.1981.tb06928.x 7348034

[51] Sima AA Brismar T. Reversible diabetic nerve dysfunction: structural correlates to electrophysiological abnormalities. Ann Neurol (1985) 18(1):21–9. doi: 10.1002/ana.410180105 3898998

[52] Sima A Lattimer SA Yagihashi S Greene DA. Axo-glial dysjunction. a novel structural lesion that accounts for poorly reversible slowing of nerve conduction in the spontaneously diabetic bio-breeding rat. J Clin Invest (1986) 77(2):474–84. doi: 10.1172/jci112326 PMC423368 3003160

[53] Sima AA Bril V Nathaniel V McEwen TA Brown MB Lattimer SA. Regeneration and repair of myelinated fibers in sural-nerve biopsy specimens from patients with diabetic neuropathy treated with sorbinil. New Engl J Med (1988) 319(9):548–55. doi: 10.1056/NEJM198809013190905 3136331

[54] Hodgkin A. A note on conduction velocity. J Physiol (1954) 125(1):221. doi: 10.1113/jphysiol.1954.sp005152 13192767 PMC1365705

[55] Sima A Renold A Shafrir E. Natural history of structural and functional alterations in diabetic BB-rat peripheral nerve. Front Diabetes Res (1988) 9(10):474–6. doi: 10.1006/mvre.1996.2002

[56] Sima A. New insights into the metabolic and molecular basis for diabetic neuropathy. Cell Mol Life Sci CMLS (2003) 60(11):2445–64. doi: 10.1007/s00018-003-3084-x PMC11138496 14625688

[57] Griffin JW Watson DF. Axonal transport in neurological disease. Ann Neurol (1988) 23(1):3–13. doi: 10.1002/ana.410230103 3278671

[58] Cheng HT Dauch JR Porzio MT Yanik BM Hsieh W Smith AG. Increased axonal regeneration and swellings in intraepidermal nerve fibers characterize painful phenotypes of diabetic neuropathy. J Pain (2013) 14(9):941–7. doi: 10.1016/j.jpain.2013.03.005 PMC3994562 23685187

[59] Gibbons C Griffin J Polydefkis M Bonyhay I Brown A Hauer P. The utility of skin biopsy for prediction of progression in suspected small fiber neuropathy. Neurology (2006) 66(2):256–8. doi: 10.1212/01.wnl.0000194314.86486.a2 16434668

[60] Ebenezer GJ McArthur JC Thomas D Murinson B Hauer P Polydefkis M. Denervation of skin in neuropathies: the sequence of axonal and schwann cell changes in skin biopsies. Brain (2007) 130(10):2703–14. doi: 10.1093/brain/awm199 17898011

[61] Chen P Piao X Bonaldo P. Role of macrophages in wallerian degeneration and axonal regeneration after peripheral nerve injury. Acta Neuropathol (2015) 130(5):605–18. doi: 10.1007/s00401-015-1482-4 26419777

[62] Mack TG Reiner M Beirowski B Mi W Emanuelli M Wagner D. Wallerian degeneration of injured axons and synapses is delayed by a Ube4b/Nmnat chimeric gene. Nat Neurosci (2001) 4(12):1199–206. doi: 10.1038/nn770 11770485

[63] Conforti L Gilley J Coleman MP. Wallerian degeneration: an emerging axon death pathway linking injury and disease. Nat Rev Neurosci (2014) 15(6):394–409. doi: 10.1038/nrn3680 24840802

[64] Gerdts J Summers DW Milbrandt J DiAntonio A. Axon self-destruction: new links among SARM1, MAPKs, and NAD+ metabolism. Neuron (2016) 89(3):449–60. doi: 10.1016/j.neuron.2015.12.023 PMC4742785 26844829

[65] Herrmann DN McDermott MP Henderson D Chen L Akowuah K Schifitto G. Epidermal nerve fiber density, axonal swellings and QST as predictors of HIV distal sensory neuropathy. Muscle Nerve (2004) 29(3):420–7. doi: 10.1002/mus.10567 14981742

[66] Lauria G Lombardi R Camozzi F Devigili G. Skin biopsy for the diagnosis of peripheral neuropathy. Histopathology (2009) 54(3):273–85. doi: 10.1111/j.1365-2559.2008.03096.x 18637969

[67] Lauria G Morbin M Lombardi R Borgna M Mazzoleni G Sghirlanzoni A. Axonal swellings predict the degeneration of epidermal nerve fibers in painful neuropathies. Neurology (2003) 61(5):631–6. doi: 10.1212/01.WNL.0000070781.92512.A4 12963753

[68] Rasband M. N. Peles E. Mechanisms of node of Ranvier assembly[J]. Nature Reviews Neuroscience (2021) 22(1):7–20. doi: 10.1038/s41583-020-00406-8 33239761

[69] Bouldin T Cavanagh J. Organophosphorous neuropathy. II. a fine-structural study of the early stages of axonal degeneration. Am J Pathol (1979) 94(2):253. doi: 10.1083/jcb.12.2.361 426028 PMC2042252

[70] Salzer JL. Polarized domains of myelinated axons. Neuron (2003) 40(2):297–318. doi: 10.1016/S0896-6273(03)00628-7 14556710

[71] Previtali SC Feltri ML Archelos JJ Quattrini A Wrabetz L Hartung H-P. Role of integrins in the peripheral nervous system. Prog Neurobiol (2001) 64(1):35–49. doi: 10.1016/S0301-0082(00)00045-9 11250061

[72] Trapp BD. Myelin-associated glycoprotein location and potential functions a. Ann New York Acad Sci (1990) 605(1):29–43. doi: 10.1111/j.1749-6632.1990.tb42378.x 1702602

[73] Jakobsen J Sidenius P. Decreased axonal flux of retrogradely transported glycoproteins in early experimental diabetes. J Neurochem (1979) 33(5):1055–60. doi: 10.1111/j.1471-4159.1979.tb05241.x 91664

[74] Jakobsen J. Early and preventable changes of peripheral nerve structure and function in insulin-deficient diabetic rats. J Neurol Neurosurg Psychiatry (1979) 42(6):509–18. doi: 10.1136/jnnp.42.6.509 PMC490254 469558

[75] Sidenius P Jakobsen J. Impaired retrograde axonal transport from a nerve crush in streptozotocin diabetic rats. Diabetologia (1980) 19(3):222–8. doi: 10.1007/BF00275273 6157594

[76] Jakobsen J. Axonal dwindling in early experimental diabetes. i. a study of cross sectioned nerves. Diabetologia (1976) 12(6):539–46. doi: 10.1007/bf01220629 137157

[77] Jakobsen J. Axonal dwindling in early experimental diabetes. II. a study of isolated nerve fibres. Diabetologia (1976) 12(6):547–53. doi: 10.1007/bf01220630 137158

[78] Kuijpers M van de Willige D Freal A Chazeau A Franker MA Hofenk J. Dynein regulator NDEL1 controls polarized cargo transport at the axon initial segment. Neuron (2016) 89(3):461–71. doi: 10.1016/j.neuron.2016.01.022 26844830

[79] Bréchet A Fache M-P Brachet A Ferracci G Baude A Irondelle M. Protein kinase CK2 contributes to the organization of sodium channels in axonal membranes by regulating their interactions with ankyrin G. J Cell Biol (2008) 183(6):1101–14. doi: 10.1083/jcb.200805169 PMC2600743 19064667

[80] Berger SL Leo-Macias A Yuen S Khatri L Pfennig S Zhang Y. Localized myosin II activity regulates assembly and plasticity of the axon initial segment. Neuron (2018) 97(3):555–570. e556. doi: 10.1016/j.neuron.2017.12.039 29395909 PMC5805619

[81] Abouelezz A Stefen H Segerstråle M Micinski D Minkeviciene R Lahti L. Tropomyosin Tpm3. 1 is required to maintain the structure and function of the axon initial segment. Iscience (2020) 23(5):101053. doi: 10.1016/j.isci.2020.101053 32344377 PMC7186529

[82] Kole MH Ilschner SU Kampa BM Williams SR Ruben PC Stuart GJ. Action potential generation requires a high sodium channel density in the axon initial segment. Nat Neurosci (2008) 11(2):178–86. doi: 10.1038/nn2040 18204443

[83] Morris RL Hollenbeck PJ. The regulation of bidirectional mitochondrial transport is coordinated with axonal outgrowth. J Cell Sci (1993) 104(3):917–27. doi: 10.1242/jcs.104.3.917 8314882

[84] Ruthel G Hollenbeck PJ. Response of mitochondrial traffic to axon determination and differential branch growth. J Neurosci (2003) 23(24):8618–24. doi: 10.1523/JNEUROSCI.23-24-08618.2003 PMC6740379 13679431

[85] Zhang CL Ho PL Kintner DB Sun D Chiu SY. Activity-dependent regulation of mitochondrial motility by calcium and Na/K-ATPase at nodes of ranvier of myelinated nerves. J Neurosci (2010) 30(10):3555–66. doi: 10.1523/JNEUROSCI.4551-09.2010 PMC3548432 20219989

[86] Errea O Moreno B Gonzalez-Franquesa A Garcia-Roves PM Villoslada P. The disruption of mitochondrial axonal transport is an early event in neuroinflammation. J Neuroinflamm (2015) 12(1):1–15. doi: 10.1186/s12974-015-0375-8 PMC4551771 26310930

[87] Waxman SG. Mechanisms of disease: sodium channels and neuroprotection in multiple sclerosis–current status. Nat Clin Pract Neurol (2008) 4(3):159–69. doi: 10.1038/ncpneuro0735 18227822

[88] Witte ME Mahad DJ Lassmann H van Horssen J. Mitochondrial dysfunction contributes to neurodegeneration in multiple sclerosis. Trends Mol Med (2014) 20(3):179187. doi: 10.1016/j.molmed.2013.11.007 24369898

[89] Xu G Pierson CR Murakawa Y Sima AA. Altered tubulin and neurofilament expression and impaired axonal growth in diabetic nerve regeneration. J Neuropathol Exp Neurol (2002) 61(2):164–75. doi: 10.1093/jnen/61.2.164 11855383

[90] Tomlinson D Fernyhough P Diemel L. Role of neurotrophins in diabetic neuropathy and treatment with nerve growth factors. Diabetes (1997) 46(Supplement_2):S43–9. doi: 10.2337/diab.46.2.S43 9285498

[91] Skundric DS Lisak RP. Role of neuropoietic cytokines in development and progression of diabetic polyneuropathy: from glucose metabolism to neurodegeneration. Exp Diabesity Res (2003) 4(4):303–12. doi: 10.1155/EDR.2003.303 PMC2478613 14668051

[92] Pekiner C Dent EW Roberts RE Meiri KF McLean WG. Altered GAP-43 immunoreactivity in regenerating sciatic nerve of diabetic rats. Diabetes (1996) 45(2):199204. doi: 10.2337/diab.45.2.199 8549865

[93] Skene J Virág I. Posttranslational membrane attachment and dynamic fatty acylation of a neuronal growth cone protein, GAP-43. J Cell Biol (1989) 108(2):613624. doi: 10.1083/jcb.108.2.613 PMC2115450 2918027

[94] Verkade P Verkleij A Gispen W Oestreicher A. Ultrastructural evidence for the lack of co-transport of b-50/GAP-43 and calmodulin in myelinated axons of the regenerating rat sciatic nerve. J Neurocytol (1996) 25(1):583–95. doi: 10.1007/BF02284826 8971638

[95] Alexander KA Cimler BM Meier KE Storm DR. Regulation of calmodulin binding to P57. a neurospecific calmodulin binding protein. J Biol Chem (1987) 262(13):6108–13. doi: 10.1016/s0021-9258(18)45544-5 2952648

[96] Alexander KA Wakim BT Doyle GS Walsh KA Storm DR. Identification and characterization of the calmodulin-binding domain of neuromodulin, a neurospecific calmodulin-binding protein. J Biol Chem (1988) 263(16):7544–9. doi: 10.1016/S0021-9258(18)68533-3 2967288

[97] Negre-Salvayre A Salvayre R Augé N Pamplona R Portero-Otin M. Hyperglycemia and glycation in diabetic complications. Antioxid Redox Signaling (2009) 11(12):30713109. doi: 10.1089/ars.2009.2484 19489690

[98] McLean WG Pekiner C Cullum NA Casson IF. Posttranslational modifications of nerve cytoskeletal proteins in experimental diabetes. Mol Neurobiol (1992) 6(2):225237. doi: 10.1007/BF02780555 1476675

[99] Hammes H-P Martin S Federlin K Geisen K Brownlee M. Aminoguanidine treatment inhibits the development of experimental diabetic retinopathy. Proc Natl Acad Sci (1991) 88(24):11555–8. doi: 10.1073/pnas.88.24.11555 PMC53174 1763069

[100] Ryle C Donaghy M. Non-enzymatic glycation of peripheral nerve proteins in human diabetics. J Neurol Sci (1995) 129(1):62–8. doi: 10.1016/0022-510X(94)00251-I 7751847

[101] Friedman M. Chemically reactive and unreactive lysine as an index of browning. Diabetes (1982) 31(Supplement_3):5–14. doi: 10.2337/diab.31.3.S5

[102] Juranek JK Geddis MS Rosario R Schmidt AM. Impaired slow axonal transport in diabetic peripheral nerve is independent of RAGE. Eur J Neurosci (2013) 38(8):3159–68. doi: 10.1111/ejn.12333 23941591

[103] Sugimoto K Yasujima M Yagihashi S. Role of advanced glycation end products in diabetic neuropathy. Curr Pharm Design (2008) 14(10):953–61. doi: 10.2174/138161208784139774 18473845

[104] Zglejc-Waszak K Mukherjee K Juranek JK. The cross-talk between RAGE and DIAPH1 in neurological complications of diabetes: A review. Eur J Neurosci (2021) 54(6):5982–99. doi: 10.1111/ejn.15433 34449932

[105] Pekiner C Cullum NA Hughes JN Hargreaves AJ Mahon J Casson IF. Glycation of brain actin in experimental diabetes. J Neurochem (1993) 61(2):436–42. doi: 10.1111/j.1471-4159.1993.tb02143.x 8336132

[106] Duran-Jimenez B Dobler D Moffatt S Rabbani N Streuli CH Thornalley PJ. Advanced glycation end products in extracellular matrix proteins contribute to the failure of sensory nerve regeneration in diabetes. Diabetes (2009) 58(12):2893–903. doi: 10.2337/db09-0320 PMC2780874 19720799

[107] Osonoi S Mizukami H Takeuchi Y Sugawa H Ogasawara S Takaku S. RAGE activation in macrophages and development of experimental diabetic polyneuropathy. JCI Insight (2022) 7(23). doi: 10.1172/jci.insight.160555 PMC9746912 36477360

[108] Cullum N Mahon J Stringer K McLean W. Glycation of rat sciatic nerve tubulin in experimental diabetes mellitus. Diabetologia (1991) 34(6):387–9. doi: 10.1007/BF00403175 1715829

[109] Janke C. The tubulin code: molecular components, readout mechanisms, and functions. J Cell Biol (2014) 206(4):461–72. doi: 10.1083/jcb.201406055 PMC4137062 25135932

[110] Keramati AR Fathzadeh M Go G-W Singh R Choi M Faramarzi S. A form of the metabolic syndrome associated with mutations in DYRK1B. N Engl J Med (2014) 370:1909–19. doi: 10.1056/NEJMoa1301824 PMC4069260 24827035

[111] Westermann S Weber K. Post-translational modifications regulate microtubule function. Nat Rev Mol Cell Biol (2003) 4(12):938–48. doi: 10.1038/nrm1260 14685172

[112] Cho Y Cavalli V. HDAC signaling in neuronal development and axon regeneration. Curr Opin Neurobiol (2014) 27:118–26. doi: 10.1016/j.conb.2014.03.008 PMC4122610 24727244

[113] Eira J Magalhães J Macedo N Pero ME Misgeld T Sousa MM. Transthyretin promotes axon growth via regulation of microtubule dynamics and tubulin acetylation. Front Cell Dev Biol (2021) 9. doi: 10.3389/fcell.2021.747699 PMC8606651 34820375

[114] Janke C Montagnac G. Causes and consequences of microtubule acetylation. Curr Biol (2017) 27(23):R1287–92. doi: 10.1016/j.cub.2017.10.044 29207274

[115] Morley SJ Qi Y Iovino L Andolfi L Guo D Kalebic N. Acetylated tubulin is essential for touch sensation in mice. Elife (2016) 5:e20813. doi: 10.7554/eLife.20813 27976998 PMC5158137

[116] Xu Z Schaedel L Portran D Aguilar A Gaillard J Marinkovich MP. Microtubules acquire resistance from mechanical breakage through intralumenal acetylation. Science (2017) 356(6335):328–32. doi: 10.1126/science.aai8764 PMC5457157 28428427

[117] Pero ME Chowdhury F Bartolini F. Role of tubulin post-translational modifications in peripheral neuropathy. Exp Neurol (2023) 360:114274. doi: 10.1016/j.expneurol.2022.114274 36379274 PMC11320756

[118] Cai D McEwen DP Martens JR Meyhofer E Verhey KJ. Single molecule imaging reveals differences in microtubule track selection between kinesin motors. PloS Biol (2009) 7(10):e1000216. doi: 10.1371/journal.pbio.1000216 19823565 PMC2749942

[119] Hammond JW Huang C-F Kaech S Jacobson C Banker G Verhey KJ. Posttranslational modifications of tubulin and the polarized transport of kinesin-1 in neurons. Mol Biol Cell (2010) 21(4):572–83. doi: 10.1091/mbc.e09-01-0044 PMC2820422 20032309

[120] Reed NA Cai D Blasius TL Jih GT Meyhofer E Gaertig J. Microtubule acetylation promotes kinesin-1 binding and transport. Curr Biol (2006) 16(21):21662172. doi: 10.1016/j.cub.2006.09.014 17084703

[121] Walter WJ Beranek V Fischermeier E Diez S. Tubulin acetylation alone does not affect kinesin-1 velocity and run length *in vitro* . (2012). doi: 10.1371/journal.pone.0042218 PMC3411631 22870307

[122] Dompierre JP Godin JD Charrin BC Cordelieres FP King SJ Humbert S. Histone deacetylase 6 inhibition compensates for the transport deficit in huntington’s disease by increasing tubulin acetylation. J Neurosci (2007) 27(13):3571–83. doi: 10.1523/JNEUROSCI.0037-07.2007 PMC6672116 17392473

[123] Tas RP Chazeau A Cloin BM Lambers ML Hoogenraad CC Kapitein LC. Differentiation between oppositely oriented microtubules controls polarized neuronal transport. Neuron (2017) 96(6):1264–1271. e1265. doi: 10.1016/j.neuron.2017.11.018 29198755 PMC5746200

[124] Abrisch RG Gumbin SC Wisniewski BT Lackner LL Voeltz GK. Fission and fusion machineries converge at ER contact sites to regulate mitochondrial morphology. J Cell Biol (2020) 219(4). doi: 10.1083/jcb.201911122 PMC7147108 32328629

[125] Balabanian L Berger CL Hendricks AG. Acetylated microtubules are preferentially bundled leading to enhanced kinesin-1 motility. Biophys J (2017) 113(7):15511560. doi: 10.1016/j.bpj.2017.08.009 PMC5627185 28978447

[126] Friedman JR Webster BM Mastronarde DN Verhey KJ Voeltz GK. ER sliding dynamics and ER–mitochondrial contacts occur on acetylated microtubules. J Cell Biol (2010) 190(3):363–75. doi: 10.1083/jcb.200911024 PMC2922647 20696706

[127] Hubert C Guardiola A Shao R. HDAC6 is a microtubule-associated deacylase. Nature (2002) 417:455–8. doi: 10.1038/417455a 12024216

[128] Rivieccio MA Brochier C Willis DE Walker BA D’Annibale MA McLaughlin K. HDAC6 is a target for protection and regeneration following injury in the nervous system. Proc Natl Acad Sci (2009) 106(46):19599–604. doi: 10.1073/pnas.0907935106 PMC2780768 19884510

[129] d’Ydewalle C Bogaert E Van Den Bosch L. HDAC 6 at the intersection of neuroprotection and neurodegeneration. Traffic (2012) 13(6):771–9. doi: 10.1111/j.1600-0854.2012.01347.x 22372633

[130] Cheng A Hou Y Mattson MP. Mitochondria and neuroplasticity. ASN Neuro (2010) 2(5):AN20100019. doi: 10.1042/AN20100019 PMC2949087 20957078

[131] Picci C Wong VS Costa CJ McKinnon MC Goldberg DC Swift M. HDAC6 inhibition promotes α-tubulin acetylation and ameliorates CMT2A peripheral neuropathy in mice. Exp Neurol (2020) 328:113281. doi: 10.1016/j.expneurol.2020.113281 32147437

[132] Sakloth F Manouras L Avrampou K Mitsi V Serafini RA Pryce KD. HDAC6-selective inhibitors decrease nerve-injury and inflammation-associated mechanical hypersensitivity in mice. Psychopharmacology (2020) 237(7):2139–49. doi: 10.1007/s00213-020-05525-9 PMC7470631 32388618

[133] Pant HC Grant P. Regulation of axonal neurofilament phosphorylation. Curr Topics Cell Regul (2001) 36:133–III. doi: 10.1016/S0070-2137(01)80006-6 10842750

[134] Tourrière H Chebli K Zekri L Courselaud B Blanchard JM Bertrand E. The RasGAP-associated endoribonuclease G3BP assembles stress granules. J Cell Biol (2003) 160(6):823–31. doi: 10.1083/jcb.200212128 PMC2173781 12642610

[135] Lee K-S Lu B. The myriad roles of miro in the nervous system: axonal transport of mitochondria and beyond. Front Cell Neurosci (2014) 8:330. doi: 10.3389/fncel.2014.00330 25389385 PMC4211407

[136] Jitprapaikulsan J Klein C Pittock SJ Gadoth A McKeon A Mills JR. Phenotypic presentations of paraneoplastic neuropathies associated with MAP1B-IgG. J Neurol Neurosurg Psychiatry (2020) 91(3):328–30. doi: 10.1136/jnnp-2019-322175 PMC7035677 31801846

[137] Ma D Connors T Nothias F Fischer I. Regulation of the expression and phosphorylation of microtubule-associated protein 1B during regeneration of adult dorsal root ganglion neurons. Neuroscience (2000) 99(1):157–70. doi: 10.1016/S0306-4522(00)00141-X 10924960

[138] Villarroel-Campos D Gonzalez-Billault C. The MAP1B case: an old MAP that is new again. Dev Neurobiol (2014) 74(10):953–71. doi: 10.1002/dneu.22178 24700609

[139] Bouquet C Ravaille-Veron M Propst F Nothias F. MAP1B coordinates microtubule and actin filament remodeling in adult mouse schwann cell tips and DRG neuron growth cones. Mol Cell Neurosci (2007) 36(2):235–47. doi: 10.1016/j.mcn.2007.07.002 17764972

[140] Nunez J Fischer I. Microtubule-associated proteins (MAPs) in the peripheral nervous system during development and regeneration. J Mol Neurosci (1997) 8(3):207–22. doi: 10.1007/BF02736834 9297633

[141] Meli R Weisová P Propst F. Repulsive axon guidance by draxin is mediated by protein kinase b (Akt), glycogen synthase kinase-3β (GSK-3β) and microtubule-associated protein 1B. PloS One (2015) 10(3):e0119524. doi: 10.1371/journal.pone.0119524 25775433 PMC4361590

[142] Hatta T Iemura S-I Ohishi T Nakayama H Seimiya H Yasuda T. Natsume T: Calpain-10 regulates actin dynamics by proteolysis of microtubuleassociated protein 1B. Sci Rep (2018) 8(1):1–9. doi: 10.1038/s41598-018-35204-x 30425305 PMC6233169

[143] Gao FJ Hebbar S Gao XA Alexander M Pandey JP Walla MD. GSK-3β phosphorylation of cytoplasmic dynein reduces Ndel1 binding to intermediate chains and alters dynein motility. Traffic (2015) 16(9):941–61. doi: 10.1111/tra.12304 PMC4543430 26010407

[144] Ogawa F Malavasi EL Crummie DK Eykelenboom JE Soares DC Mackie S. DISC1 complexes with TRAK1 and Miro1 to modulate anterograde axonal mitochondrial trafficking. Hum Mol Genet (2014) 23(4):906–19. doi: 10.1093/hmg/ddt485 PMC3900104 24092329

[145] Dolma K Iacobucci GJ Hong Zheng K Shandilya J Toska E White JA. Presenilin influences glycogen synthase kinase-3 β (GSK-3β) for kinesin1 and dynein function during axonal transport. Hum Mol Genet (2014) 23(5):1121–33. doi: 10.1093/hmg/ddt505 24105467

[146] Llorens-Martin M Lopez-Domenech G Soriano E Avila J. GSK3β is involved in the relief of mitochondria pausing in a tau-dependent manner. PloS One (2011) 6(11):e27686. doi: 10.1371/journal.pone.0027686 22110721 PMC3215736

[147] Fernyhough P Gallagher A Averill SA Priestley JV Hounsom L Patel J. Aberrant neurofilament phosphorylation in sensory neurons of rats with diabetic neuropathy. Diabetes (1999) 48(4):881–9. doi: 10.2337/diabetes.48.4.881 10102707

[148] Mohiuddin L Fernyhough P Tomlinson DR. Reduced levels of mRNA encoding endoskeletal and growth-associated proteins in sensory ganglia in experimental diabetes. Diabetes (1995) 44(1):25–30. doi: 10.2337/diab.44.1.25 7813810

[149] Yagihashi S Kamijo M Watanabe K. Reduced myelinated fiber size correlates with loss of axonal neurofilaments in peripheral nerve of chronically streptozotocin diabetic rats. Am J Pathol (1990) 136(6):1365. doi: 10.1177/107385849500100204 2141449 PMC1877565

[150] Pekiner C McLean WG. Neurofilament protein phosphorylation in spinal cord of experimentally diabetic rats. J Neurochem (1991) 56(4):1362–7. doi: 10.1111/j.1471-4159.1991.tb11433.x 1848279

[151] Kamiya H Zhang W Sima AA. Dynamic changes of neuroskeletal proteins in DRGs underlie impaired axonal maturation and progressive axonal degeneration in type 1 diabetes. Exp Diabetes Res (2009) 2009. doi: 10.1155/2009/793281 PMC2761046 19834568

[152] Hellweg R Raivich G Hartung H-D Hock C Kreutzberg GW. Axonal transport of endogenous nerve growth factor (NGF) and NGF receptor in experimental diabetic neuropathy. Exp Neurol (1994) 130(1):24–30. doi: 10.1006/exnr.1994.1181 7821393

[153] Fernyhough P Schmidt RE. Neurofilaments in diabetic neuropathy. Int Rev Neurobiol (2002) 50:115–44. doi: 10.1016/S0074-7742(02)50075-1 12198808

[154] Morfini GA You Y-M Pollema SL Kaminska A Liu K Yoshioka K. Pathogenic huntingtin inhibits fast axonal transport by activating JNK3 and phosphorylating kinesin. Nat Neurosci (2009) 12(7):864–71. doi: 10.1038/nn.2346 PMC2739046 19525941

[155] Hirokawa N Niwa S Tanaka Y. Molecular motors in neurons: transport mechanisms and roles in brain function, development, and disease. Neuron (2010) 68(4):610–38. doi: 10.1016/j.neuron.2010.09.039 21092854

[156] Fernyhough P Tomlinson DR. The therapeutic potential of neurotrophins for the treatment of diabetic neuropathy. Diabetes Rev (1999) 7(4):300–11. doi: 10.1007/978-1-4612-1816-6_8

[157] Middlemas A Delcroix JD Sayers N Tomlinson D Fernyhough P. Enhanced activation of axonally transported stress-activated protein kinases in peripheral nerve in diabetic neuropathy is prevented by neurotrophin-3. Brain (2003) 126(7):1671–82. doi: 10.1093/brain/awg150 12805110

[158] Park C-J Seo Y-S. Heat shock proteins: a review of the molecular chaperones for plant immunity. Plant Pathol J (2015) 31(4):323. doi: 10.5423/PPJ.RW.08.2015.0150 26676169 PMC4677741

[159] Muchowski PJ Wacker JL. Modulation of neurodegeneration by molecular chaperones. Nat Rev Neurosci (2005) 6(1):11–22. doi: 10.1038/nrn1587 15611723

[160] Adalbert R Kaieda A Antoniou C Loreto A Yang X Gilley J. Novel HDAC6 inhibitors increase tubulin acetylation and rescue axonal transport of mitochondria in a model of charcot–Marie–Tooth type 2F. ACS Chem Neurosci (2019) 11(3):258–67. doi: 10.1021/acschemneuro.9b00338.s001 PMC7614726 31845794

[161] Benn SC Perrelet D Kato AC Scholz J Decosterd I Mannion RJ. Hsp27 upregulation and phosphorylation is required for injured sensory and motor neuron survival. Neuron (2002) 36(1):45–56. doi: 10.1016/S0896-6273(02)00941-8 12367505

[162] Dodge ME Wang J Guy C Rankin S Rahimtula M Mearow KM. Stress-induced heat shock protein 27 expression and its role in dorsal root ganglion neuronal survival. Brain Res (2006) 1068(1):34–48. doi: 10.1016/j.brainres.2005.11.008 16376863

[163] Zochodne DW Verge VM Cheng C Sun H Johnston J. Does diabetes target ganglion neurones? progressive sensory neurone involvement in long-term experimental diabetes. Brain (2001) 124(11):2319–34. doi: 10.1093/brain/124.11.2319 11673332

[164] Dunlop ME Muggli EE. Small heat shock protein alteration provides a mechanism to reduce mesangial cell contractility in diabetes and oxidative stress. Kidney Int (2000) 57(2):464–75. doi: 10.1046/j.1523-1755.2000.00866.x 10652023

[165] Joussen AM Huang S Poulaki V Camphausen K Beecken W-D Kirchhof B. *In vivo* retinal gene expression in early diabetes. Invest Ophthalmol Visual Sci (2001) 42(12):3047–57. doi: 10.1167/iovs.06-0723 11687554

[166] Park HK Park E-C Bae SW Park MY Kim SW Yoo HS. Expression of heat shock protein 27 in human atherosclerotic plaques and increased plasma level of heat shock protein 27 in patients with acute coronary syndrome. Circulation (2006) 114(9):886–93. doi: 10.1161/CIRCULATIONAHA.105.541219 16923754

[167] Lewis SE Mannion RJ White FA Coggeshall RE Beggs S Costigan M. A role for HSP27 in sensory neuron survival. J Neurosci (1999) 19(20):8945–53. doi: 10.1523/JNEUROSCI.19-20-08945.1999 PMC6782783 10516313

[168] Ma CHE Omura T Cobos EJ Latrémolière A Ghasemlou N Brenner GJ. Accelerating axonal growth promotes motor recovery after peripheral nerve injury in mice. J Clin Invest (2011) 121(11). doi: 10.1172/JCI58675 PMC3223863 21965333

[169] Kobayashi M Zochodne DW. Diabetic neuropathy and the sensory neuron: new aspects of pathogenesis and their treatment implications. J Diabetes Invest (2018) 9(6):1239–54. doi: 10.1111/jdi.12833 PMC6215951 29533535

[170] Williams KL Rahimtula M Mearow KM. Hsp27 and axonal growth in adult sensory neurons *in vitro* . BMC Neurosci (2005) 6(1):1–17. doi: 10.1016/j.neuroscience.2004.04.004 15819993 PMC1087488

[171] Korngut L Ma CHE Martinez J Toth C Guo G Singh V. Overexpression of human HSP27 protects sensory neurons from diabetes. Neurobiol Dis (2012) 47(3):436–43. doi: 10.1016/j.nbd.2012.04.017 PMC3392489 22569359

[172] Gruden G Bruno G Chaturvedi N Burt D Schalkwijk C Pinach S. Serum heat shock protein 27 and diabetes complications in the EURODIAB prospective complications study: a novel circulating marker for diabetic neuropathy. Diabetes (2008) 57(7):1966–70. doi: 10.2337/db08-0009 PMC2453614 18390793

[173] Pourhamidi K Skärstrand H Dahlin LB Rolandsson O. HSP27 concentrations are lower in patients with type 1 diabetes and correlate with large nerve fiber dysfunction. Diabetes Care (2014) 37(3):e49–50. doi: 10.2337/dc13-1780 24558083

[174] Almeida-Souza L Asselbergh B d’Ydewalle C Moonens K Goethals S De Winter V. Small heat-shock protein HSPB1 mutants stabilize microtubules in charcot-Marie-Tooth neuropathy. J Neurosci (2011) 31(43):15320–8. doi: 10.1523/JNEUROSCI.3266-11.2011 PMC6703512 22031878

[175] Uribe JS Schwab F Mundis GM Xu DS Januszewski J Kanter AS. The comprehensive anatomical spinal osteotomy and anterior column realignment classification: Presented at the 2018 AANS/CNS joint section on disorders of the spine and peripheral nerves. J Neurosurg: Spine (2018) 29(5):565–75. doi: 10.3171/2018.4.SPINE171206 30141765

[176] Prior R Van Helleputte L Benoy V Van Den Bosch L. Defective axonal transport: a common pathological mechanism in inherited and acquired peripheral neuropathies. Neurobiol Dis (2017) 105:300–20. doi: 10.1016/j.nbd.2017.02.009 28238949

[177] Kim J-Y Woo S-Y Hong YB Choi H Kim J Choi H. HDAC6 inhibitors rescued the defective axonal mitochondrial movement in motor neurons derived from the induced pluripotent stem cells of peripheral neuropathy patients with HSPB1 mutation. Stem Cells Int (2016) 2016. doi: 10.1155/2016/9475981 PMC5220520 28105056

[178] Zimmerman M Enes SR Skärstrand H Pourhamidi K Gottsäter A Wollmer P. Temporal trend of autonomic nerve function and HSP27, MIF and PAI-1 in type 1 diabetes. J Clin Trans Endocrinol (2017) 8:15–21. doi: 10.1016/j.jcte.2017.03.001 PMC5651332 29067254

[179] Schroer TA. Dynactin. Annu Rev Cell Dev Biol (2004) 20:759. doi: 10.1146/annurev.cellbio.20.012103.094623 15473859

[180] Brownlees J Ackerley S Grierson AJ Jacobsen NJ Shea K Anderton BH. Charcot–Marie–Tooth disease neurofilament mutations disrupt neurofilament assembly and axonal transport. Hum Mol Genet (2002) 11(23):2837–44. doi: 10.1093/hmg/11.23.2837 12393795

[181] Sheng Z-H. Mitochondrial trafficking and anchoring in neurons: new insight and implications. J Cell Biol (2014) 204(7):1087–98. doi: 10.1083/jcb.201312123 PMC3971748 24687278

[182] Miki H Setou M Kaneshiro K Hirokawa N. All kinesin superfamily protein, KIF, genes in mouse and human. Proc Natl Acad Sci (2001) 98(13):70047011. doi: 10.1073/pnas.111145398 PMC34614 11416179

[183] Kanai Y Okada Y Tanaka Y Harada A Terada S Hirokawa N. KIF5C, a novel neuronal kinesin enriched in motor neurons. J Neurosci (2000) 20(17):6374–84. doi: 10.1523/JNEUROSCI.20-17-06374.2000 PMC6772948 10964943

[184] Rahmati M Gharakhanlou R Movahedin M Mowla SJ Khazani A Fouladvand M. Treadmill training modifies KIF5B motor protein in the STZ-induced diabetic rat spinal cord and sciatic nerve. Arch Iranian Med (2015) 18(2):0–0. doi: 10.1213/ane.0000000000000799 25644797

[185] Pesaresi M Giatti S Spezzano R Romano S Diviccaro S Borsello T. Axonal transport in a peripheral diabetic neuropathy model: sex-dimorphic features. Biol Sex Dif (2018) 9(1):1–14. doi: 10.1186/s13293-018-0164-z PMC5775621 29351809

[186] Maximino JR de Oliveira GP Alves CJ Chadi G. Deregulated expression of cytoskeleton related genes in the spinal cord and sciatic nerve of presymptomatic SOD1G93A amyotrophic lateral sclerosis mouse model. Front Cell Neurosci (2014) 8:148. doi: 10.3389/fncel.2014.00148 24904291 PMC4033281

[187] Medori R Jenich H Autilio-Gambetti L Gambetti P. Experimental diabetic neuropathy: similar changes of slow axonal transport and axonal size in different animal models. J Neurosci (1988) 8(5):1814–21. doi: 10.1523/JNEUROSCI.08-05-01814.1988 PMC6569209 3367221

[188] Larsen JR Sidenius P. Slow axonal transport of structural polypeptides in rat, early changes in streptozocin diabetes, and effect of insulin treatment. J Neurochem (1989) 52(2):390–401. doi: 10.1111/j.1471-4159.1989.tb09134.x 2463334

[189] Xiao Q Hu X Wei Z Tam KY. Cytoskeleton molecular motors: structures and their functions in neuron. Int J Biol Sci (2016) 12(9):1083. doi: 10.7150/ijbs.15633 27570482 PMC4997052

[190] Wang L Brown A. A hereditary spastic paraplegia mutation in kinesin-1A/KIF5A disrupts neurofilament transport. Mol Neurodegeneration (2010) 5(1):1–13. doi: 10.1186/1750-1326-5-52 PMC3000839 21087519

[191] Tanaka Y Niwa S Dong M Farkhondeh A Wang L Zhou R. The molecular motor KIF1A transports the TrkA neurotrophin receptor and is essential for sensory neuron survival and function. Neuron (2016) 90(6):1215–29. doi: 10.1016/j.neuron.2016.05.002 27263974

[192] Yonekawa Y Harada A Okada Y Funakoshi T Kanai Y Takei Y. Defect in synaptic vesicle precursor transport and neuronal cell death in KIF1A motor protein–deficient mice. J Cell Biol (1998) 141(2):431–41. doi: 10.1083/jcb.141.2.431 PMC2148442 9548721

[193] Bronson SC Suresh E Kumar SSAS Mythili C Shanmugam A. A novel synergistic association of variants in PTRH2 and KIF1A relates to a syndrome of hereditary axonopathy, outer hair cell dysfunction, intellectual disability, pancreatic lipomatosis, diabetes, cerebellar atrophy, and vertebral artery hypoplasia. Cureus (2021) 13(2). doi: 10.7759/cureus.13174 PMC7939034 33717719

[194] Karle KN Möckel D Reid E Schöls L. Axonal transport deficit in a KIF5A–/–mouse model. Neurogenetics (2012) 13(2):169–79. doi: 10.1007/s10048-012-0324-y PMC3332386 22466687

[195] Aaberg ML Burch DM Hud ZR Zacharias MP. Gender differences in the onset of diabetic neuropathy. J Diabetes Complications (2008) 22(2):83–7. doi: 10.1016/j.jdiacomp.2007.06.009 18280437

[196] Kiziltan ME Benbir G. Clinical and electrophysiological differences in male and female patients with diabetic foot. Diabetes Res Clin Pract (2008) 79(1):e17–8. doi: 10.1016/j.diabres.2007.07.013 17764777

[197] Kiziltan ME Gunduz A Kiziltan G Akalin MA Uzun N. Peripheral neuropathy in patients with diabetic foot ulcers: clinical and nerve conduction study. J Neurol Sci (2007) 258(1-2):75–9. doi: 10.1016/j.jns.2007.02.028 17399742

[198] Joseph E Levine J. Sexual dimorphism in the contribution of protein kinase c isoforms to nociception in the streptozotocin diabetic rat. Neuroscience (2003) 120(4):907–13. doi: 10.1016/S0306-4522(03)00400-7 12927197

[199] O’Brien PD Hur J Robell NJ Hayes JM Sakowski SA Feldman EL. Gender-specific differences in diabetic neuropathy in BTBR ob/ob mice. J Diabetes Complications (2016) 30(1):30–7. doi: 10.1016/j.jdiacomp.2015.09.018 PMC4698064 26525588

[200] Ye L Kleiner S Wu J Sah R Gupta RK Banks AS. TRPV4 is a regulator of adipose oxidative metabolism, inflammation, and energy homeostasis. Cell (2012) 151(1):96–110. doi: 10.1016/j.cell.2012.08.034 23021218 PMC3477522

[201] Hammerschlag R Dravid AR Chiu AY. Mechanism of axonal transport: a proposed role for calcium ions. Science (1975) 188(4185):273–5. doi: 10.1126/science.47182 47182

[202] Breuer A Atkinson M. Calcium dependent modulation of fast axonal transport. Cell Calcium (1988) 9(5-6):293–301. doi: 10.1016/0143-4160(88)90010-3 2465090

[203] Del Arco A Contreras L Pardo B Satrustegui J. Calcium regulation of mitochondrial carriers. Biochim Biophys Acta (BBA) Mol Cell Res (2016) 1863(10):2413–21. doi: 10.1016/j.bbamcr.2016.03.024 27033520

[204] Jeyaraju DV Cisbani G Pellegrini L. Calcium regulation of mitochondria motility and morphology. Biochim Biophys Acta (BBA) Bioenergetics (2009) 1787(11):1363–73. doi: 10.1016/j.bbabio.2008.12.005 19138660

[205] Mironov SL Ivannikov MV Johansson M. [Ca2+] i signaling between mitochondria and endoplasmic reticulum in neurons is regulated by microtubules: from mitochondrial permeability transition pore to Ca2+-induced Ca2+ release. J Biol Chem (2005) 280(1):715–21. doi: 10.1074/jbc.M409819200 15516333

[206] Wang X Schwarz TL. The mechanism of Ca2+-dependent regulation of kinesin-mediated mitochondrial motility. Cell (2009) 136(1):163–74. doi: 10.1016/j.cell.2008.11.046 PMC2768392 19135897

[207] Suzuki M Hirao A Mizuno A. Microfilament-associated protein 7 increases the membrane expression of transient receptor potential vanilloid 4 (TRPV4). J Biol Chem (2003) 278(51):51448–53. doi: 10.1074/jbc.M308212200 14517216

[208] Barlan K Lu W Gelfand VI. The microtubule-binding protein ensconsin is an essential cofactor of kinesin-1. Curr Biol (2013) 23(4):317–22. doi: 10.1016/j.cub.2013.01.008 PMC3580027 23394833

[209] Sung H-H Telley IA Papadaki P Ephrussi A Surrey T Rørth P. Drosophila ensconsin promotes productive recruitment of kinesin-1 to microtubules. Dev Cell (2008) 15(6):866–76. doi: 10.1016/j.devcel.2008.10.006 19081075

[210] Matsumura Y Yokoyama Y Hirakawa H Shigeto T Futagami M Mizunuma H. The prophylactic effects of a traditional Japanese medicine, goshajinkigan, on paclitaxelinduced peripheral neuropathy and its mechanism of action. Mol Pain (2014) 10(1):1–8. doi: 10.1186/1744-8069-10-61 25240613 PMC4176860

[211] Dias FC Alves VS Matias DO Figueiredo CP Miranda ALP Passos GF. The selective TRPV4 channel antagonist HC-067047 attenuates mechanical allodynia in diabetic mice. Eur J Pharmacol (2019) 856:172408. doi: 10.1016/j.ejphar.2019.172408 31129158

[212] Goswami C Kuhn J Heppenstall PA Hucho T. Importance of non-selective cation channel TRPV4 interaction with cytoskeleton and their reciprocal regulations in cultured cells. PloS One (2010) 5(7):e11654. doi: 10.1371/journal.pone.0011654 20657843 PMC2906515

[213] Cashman CR Höke A. Mechanisms of distal axonal degeneration in peripheral neuropathies. Neurosci Lett (2015) 596:33–50. doi: 10.1016/j.neulet.2015.01.048 25617478 PMC4428955

[214] Fang C Bourdette D Banker G. Oxidative stress inhibits axonal transport: implications for neurodegenerative diseases. Mol Neurodegeneration (2012) 7(1):1–13. doi: 10.1186/1750-1326-7-29 PMC3407799 22709375

[215] Zhou T Lee A Lo ACY Kwok JSWJ. Diabetic corneal neuropathy: pathogenic mechanisms and therapeutic strategies. Front Pharmacol (2022) 13. doi: 10.3389/fphar.2022.816062 PMC8905431 35281903

[216] Carrington A Litchfield J. The aldose reductase pathway and nonenzymatic glycation in the pathogenesis of diabetic neuropathy: a critical review for the end of the 20th century. Diabetes Rev (1999) 7(4):275–99. doi: 10.5353/th_b3637119

[217] Dahlin L Archer D McLean W. Treatment with an aldose reductase inhibitor can reduce the susceptibility of fast axonal transport following nerve compression in the streptozotocin-diabetic rat. Diabetologia (1987) 30(6):414–8. doi: 10.1007/BF00292544 2445613

[218] Greene DA Lattimer SA Sima AA. Are disturbances of sorbitol, phosphoinositide, and na+-K+-ATPase regulation involved in pathogenesis of diabetic neuropathy? Diabetes (1988) 37(6):688–93. doi: 10.2337/diabetes.37.6.688 2838351

[219] Fink DJ Purkiss D Mata M. Alterations in retrograde axonal transport in streptozocininduced diabetic rats. Diabetes (1987) 36(9):996–1000. doi: 10.2337/diab.36.9.996 2440748

[220] Tomlinson DR Moriarty RJ Mayer JH. Prevention and reversal of defective axonal transport and motor nerve conduction velocity in rats with experimental diabetes by treatment with the aldose reductase inhibitor sorbinil. Diabetes (1984) 33(5):470–6. doi: 10.2337/diab.33.5.470 6202576

[221] Boden G. Free fatty acids, insulin resistance, and type 2 diabetes mellitus. Proc Assoc Am Physicians (1999) 111(3):241–8. doi: 10.1046/j.1525-1381.1999.99220.x 10354364

[222] Rumora AE Lentz SI Hinder LM Jackson SW Valesano A Levinson GE. Dyslipidemia impairs mitochondrial trafficking and function in sensory neurons. FASEB J (2018) 32(1):195–207. doi: 10.1096/fj.201700206R 28904018 PMC6191072

[223] Rumora AE LoGrasso G Haidar JA Dolkowski JJ Lentz SI Feldman EL. Chain length of saturated fatty acids regulates mitochondrial trafficking and function in sensory neurons. J Lipid Res (2019) 60(1):58–70. doi: 10.1194/jlr.M086843 30442656 PMC6314260

[224] Schönfeld P Wojtczak L. Short-and medium-chain fatty acids in energy metabolism: the cellular perspective. J Lipid Res (2016) 57(6):943–54. doi: 10.1194/jlr.R067629 PMC4878196 27080715

[225] Papamandjaris AA MacDougall DE Jones PJ. Medium chain fatty acid metabolism and energy expenditure: obesity treatment implications. Life Sci (1998) 62(14):1203–15. doi: 10.1016/S0024-3205(97)01143-0 9570335

[226] O’Brien PD Guo K Eid SA Rumora AE Hinder LM Hayes JM. Integrated lipidomic and transcriptomic analyses identify altered nerve triglycerides in mouse models of prediabetes and type 2 diabetes. Dis Models Mech (2020) 13(2):dmm042101. doi: 10.1242/dmm.042101 PMC6994925 31822493

[227] Tang H-Y Jiang A-J Ma J-L Wang F-J Shen G-M. Understanding the signaling pathways related to the mechanism and treatment of diabetic peripheral neuropathy. Endocrinology (2019) 160(9):2119–27. doi: 10.1210/en.2019-00311 31318414

[228] Rumora AE LoGrasso G Hayes JM Mendelson FE Tabbey MA Haidar JA. The divergent roles of dietary saturated and monounsaturated fatty acids on nerve function in murine models of obesity. J Neurosci (2019) 39(19):37703781. doi: 10.1523/JNEUROSCI.3173-18.2019 PMC6510336 30886017

[229] Powell H. Pathology of diabetic neuropathy: new observations, new hypotheses. Lab Invest (1983) 49(5):515–8. doi: 10.1002/0470862092.d0906 6688840

[230] Rydevik B Lundborg G Bagge U. Effects of graded compression on intraneural blood flow: an *in vivo* study on rabbit tibial nerve. J Handb Surg (1981) 6(1):3–12. doi: 10.1016/S0363-5023(81)80003-2 7204915

[231] Lundborg G Myers R Powell H. Nerve compression injury and increased endoneurial fluid pressure: a” miniature compartment syndrome”. J Neurol Neurosurg Psychiatry (1983) 46(12):1119–24. doi: 10.1136/jnnp.46.12.1119 PMC491778 6663311

[232] Dahlin L Meiri KF McLean W Rydevik B Sjöstran J. Effects of nerve compression on fast axonal transport in streptozotocin-induced diabetes mellitus. Diabetologia (1986) 29(3):181–5. doi: 10.1007/BF02427090 2422081

[233] Mayer J Tomlinson D. Prevention of defects of axonal transport and nerve conduction velocity by oral administration of myo-inositol or an aldose reductase inhibitor in streptozotocin-diabetic rats. Diabetologia (1983) 25(5):433–8. doi: 10.1007/BF00282524 6197336

[234] Tomlinson DR Mayer JH. Reversal of deficits in axonal transport and nerve conduction velocity by treatment of streptozotocin-diabetic rats with myo-inositol. Exp Neurol (1985) 89(2):420–7. doi: 10.1016/0014-4886(85)90101-3 2410289

[235] Tomlinson DR Holmes PR Mayer JH. Reversal, by treatment with an aldose reductase inhibitor, of impaired axonal transport and motor nerve conduction velocity in experimental diabetes mellitus. Neurosci Lett (1982) 31(2):189–93. doi: 10.1016/0304-3940(82)90115-X 6182509

[236] Kim J Yokoyama K Araki S. The effects of ginkgo biloba extract (GBe) on axonal transport, microvasculature and morphology of sciatic nerve in streptozotocin-induced diabetic rats. Environ Health Prev Med (2001) 5(2):53–9. doi: 10.1007/bf02932004 PMC2723546 21432198

[237] Khazani A Gharakhanlou R Kordi M Movahedian M Jahani Golbar Sh RM. The effect of endurance training on dynein motor protein expression in wistar male rats sciatic nerves with diabetic neuropathy. Hormozgan Med J (2017) 21(1):10–9. doi: 10.18869/acadpub.hmj.21.1.10

[238] Moghadam Z Rezaee Shirazi R Shariatzadeh Joneydi M Asgharpour H Rahmati M. Increased levels of spinal cord KIF1B protein in healthy and diabetic neuropathic wistar rats with in adaptation to aerobic training (KIF1B changes in sensory neurons after exercise). Jundishapur Sci Med J (2021) 20(4):346–55. doi: 10.32598/JSMJ.20.4.2424

[239] Golbar SJ Gharekhanlu R Kordi MR Khazani A. Effects of endurance exercise training on kinesin-5 and dynein motor proteins in sciatic nerves of male wistar rats with diabetic neuropathy. Int J Sport Stud Health (2018) 1(1). doi: 10.5812/intjssh.67758

[240] Calcutt N Tomlinson D Willars G. Ganglioside treatment of streptozotocin-diabetic rats prevents defective axonal transport of 6-phosphofructokinase activity. J Neurochem (1988) 50(5):1478–83. doi: 10.1111/j.1471-4159.1988.tb03033.x 2452237

[241] Figliomeni B Bacci B Panozzo C Fogarolo F Triban C Fiori MG. Experimental diabetic neuropathy: effect of ganglioside treatment on axonal transport of cytoskeletal proteins. Diabetes (1992) 41(7):866–71. doi: 10.2337/diab.41.7.866 1377137

[242] Marini P Vitadello M Bianchi R Triban C Gorio A. Impaired axonal transport of acetylcholinesterase in the sciatic nerve of alloxan-diabetic rats: effect of ganglioside treatment. Diabetologia (1986) 29(4):254–8. doi: 10.1007/BF00454886 2423405

[243] Wegener OH. Urinary bladder[M]//Whole Body Computerized Tomography. (Karger Publishers) (1983), 265–8.

[244] Yagihashi S Kamijo M Baba M Yagihashi N Nagai K. Effect of aminoguanidine on functional and structural abnormalities in peripheral nerve of STZ-induced diabetic rats. Diabetes (1992) 41(1):47–52. doi: 10.2337/diab.41.1.47 1727739

[245] Sharma A. Animal models: pathologyand pathophysiology. Diabetic Neuropathy (1987) 237–52. 10.1055/b-0034-83061

[246] Jakobsen J Sidenius P. Decreased axonal transport of structural proteins in streptozotocin diabetic rats. J Clin Invest (1980) 66(2):292–7. doi: 10.1172/JCI109856 PMC371710 6156952

[247] Hoffman PN Griffin JW Price DL. Control of axonal caliber by neurofilament transport. J Cell Biol (1984) 99(2):705–14. doi: 10.1083/jcb.99.2.705 PMC2113274 6204997

[248] Williams KL Mearow KM. Phosphorylation status of heat shock protein 27 influences neurite growth in adult dorsal root ganglion sensory neurons *in vitro* . J Neurosci Res (2011) 89(8):1160–72. doi: 10.1002/jnr.22634 21638305

[249] During RL Gibson BG Li W Bishai EA Sidhu GS Landry J. Anthrax lethal toxin paralyzes actin-based motility by blocking Hsp27 phosphorylation. EMBO J (2007) 26(9):2240–50. doi: 10.1038/sj.emboj.7601687 PMC1864983 17446863

[250] Pichon S Bryckaert M Berrou E. Control of actin dynamics by p38 MAP kinase–Hsp27 distribution in the lamellipodium of smooth muscle cells. J Cell Sci (2004) 117(12):2569–77. doi: 10.1242/jcs.01110 15128872

[251] Mizutani K Miki H He H Maruta H Takenawa T. Essential role of neural WiskottAldrich syndrome protein in podosome formation and degradation of extracellular matrix in src-transformed fibroblasts. Cancer Res (2002) 62(3):669–74. doi: 10.1242/jcs.116624 11830518

[252] Cernea S Raz I. Management of diabetic neuropathy. Metabolism (2021) 123:154867. doi: 10.1016/j.metabol.2021.154867 34411554

[253] Krukowski K Ma J Golonzhka O Laumet GO Gutti T Van Duzer JH. HDAC6 inhibition effectively reverses chemotherapyinduced peripheral neuropathy. Pain (2017) 158(6):1126. doi: 10.3410/f.727378020.793571728 28267067 PMC5435512

[254] Ma J Trinh RT Mahant ID Peng B Matthias P Heijnen CJ. Cell-specific role of HDAC6 in chemotherapy-induced mechanical allodynia and loss of intraepidermal nerve fibers. Pain (2019) 160(12):2877. doi: 10.1016/j.jpain.2019.02.065 31356453 PMC6856416

[255] Benoy V Vanden Berghe P Jarpe M Van Damme P Robberecht W Van Den Bosch L. Development of improved HDAC6 inhibitors as pharmacological therapy for axonal charcot–Marie–Tooth disease. Neurotherapeutics (2017) 14(2):417–28. doi: 10.1007/s13311-016-0501-z PMC5398982 27957719

